# Loss of PIKfyve drives the spongiform degeneration in prion diseases

**DOI:** 10.15252/emmm.202114714

**Published:** 2021-07-22

**Authors:** Asvin K K Lakkaraju, Karl Frontzek, Emina Lemes, Uli Herrmann, Marco Losa, Rajlakshmi Marpakwar, Adriano Aguzzi

**Affiliations:** ^1^ Institute of Neuropathology University of Zurich Zürich Switzerland

**Keywords:** neurodegeneration, palmitoylation, prion, spongiosis, unfolded protein response, Neuroscience, Organelles

## Abstract

Brain‐matter vacuolation is a defining trait of all prion diseases, yet its cause is unknown. Here, we report that prion infection and prion‐mimetic antibodies deplete the phosphoinositide kinase PIKfyve—which controls endolysosomal maturation—from mouse brains, cultured cells, organotypic brain slices, and brains of Creutzfeldt‐Jakob disease victims. We found that PIKfyve is acylated by the acyltransferases zDHHC9 and zDHHC21, whose juxtavesicular topology is disturbed by prion infection, resulting in PIKfyve deacylation and rapid degradation, as well as endolysosomal hypertrophy and activation of TFEB‐dependent lysosomal enzymes. A protracted unfolded protein response (UPR), typical of prion diseases, also induced PIKfyve deacylation and degradation. Conversely, UPR antagonists restored PIKfyve levels in prion‐infected cells. Overexpression of zDHHC9 and zDHHC21, administration of the antiprion polythiophene LIN5044, or supplementation with the PIKfyve reaction product PI(3,5)P_2_ suppressed prion‐induced vacuolation and restored lysosomal homeostasis. Thus, PIKfyve emerges as a central mediator of vacuolation and neurotoxicity in prion diseases.

## Graphical abstract

The phosphoinositide kinase PIKfyve is identified as the driver of brain vacuolation (spongiosis) in prion diseases. A hierarchy of events, starting the delocalization of PIKfyve‐specific acyltransferases and culminating in the enlargement of endolysosomes underlying spongiosis, is established.

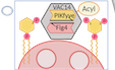

**The paper explained**

**Problem**

Intraneuronal vacuoles (“spongiosis”) are a striking feature of prion diseases. Although the appearance of these vacuoles is highly characteristic and almost unique, it is unknown whether vacuolation represents a downstream event of toxicity or conversely a driver of toxicity. If vacuolation is the proximal cause of neuronal death, its prevention may be therapeutically useful in prion diseases. However, none of that can be tested unless we understand the events leading to spongiosis—all of which are unknown thus far.

**Results**

Loss of PIKfyve, an inositide kinase localized on the cytosolic face of late endosomal membranes, leads to intracellular vacuoles. We were surprised to discover that PIKfyve is depleted in prion infections. Digging deeper, we found that PIKfyve is normally acylated and that endoplasmic reticulum stress induces its deacylation. We also discovered the two enzymes responsible for PIKfyve acylation. Deacylation destabilizes PIKfyve and causes its depletion in prion infections. Finally, the alterations in lysosomal functioning caused by PIKfyve depletion were rescued by supplying a chemical analogue of phosphoinositol‐3,5‐diphospate, the PIKfyve reaction product, or by overexpression of the acyltransferases regulating PIKfyve acylation.

**Impact**

We have elucidated the cascade of molecular events that is induced by prion infections and results in spongiosis, one of the most prominent and universal characteristics of prion diseases. Future studies will use high‐throughput forward genetics to identify those components that are still unknown. Excitingly, several components of the pathways delineated here are enzymes and therefore, in principle, druggable. As a long‐term perspective, we shall aim to test the hypothesis that manipulating PIKfyve, its acylation state, and its reaction products may provide a viable entry point to modify the course of prion diseases and/or to alleviate the symptoms of these hitherto incurable pathologies.

## Introduction

The self‐sustaining recruitment of the cellular prion protein PrP^C^ into its aggregated conformer PrP^Sc^ is the basis of prion transmissibility and neuroinvasion. The ablation of PrP^C^ prevents prion propagation (Bueler *et al*, 1993) and toxicity (Brandner *et al*, 1996). While these phenomena have been extensively studied, little is known about how PrP^Sc^ leads to neurodegeneration. The examination of prion‐infected human, ruminant, and rodent brains invariably reveals prominent intracellular vacuolation conferring a foamy appearance (spongiosis) to brain tissue. During disease progression, intraneuronal vacuoles gradually coalesce into optically transparent cavities that eventually replace much of the cortical gray matter. The ubiquity of spongiosis in prion diseases suggests that it is intimately linked to the mechanism of disease. However, little is known about the molecular drivers of spongiogenesis (Aguzzi *et al*, 2008).

The spongiosis caused by *PRNP* mutations linked to familial prion diseases was attributed to the E2 ubiquitin ligase Mahogunin ring finger‐1 (MGRN1) (Chakrabarti & Hegde, 2009). MGRN1 appears to interact with an improperly folded and processed form of PrP^C^ which accumulates in the cytosol (cyPrP) of mice expressing PrP^C^ mutants linked to genetic forms of Creutzfeldt‐Jakob disease (gCJD). This was reported to sequester MGRN1, thereby preventing it from ubiquitinating other substrates. The retention of MGRN1 by cyPrP may mimic MGRN1 depletion, which was previously shown to result in CNS vacuolation similar to the spongiform phenotype observed in prion infections (He *et al*, 2003a). However, transgenic mice expressing high or low levels of MGRN1 showed no difference in the onset, progression, and histopathology of the disease after prion infection (Gunn & Carlson, 2013; Silvius *et al*, 2013). Conversely, mice overexpressing a cytosolic form of PrP do not show spongiosis (Norstrom *et al*, 2007). Therefore, depletion of MGRN1 is not the sole cause for the phenotype observed in gCJD and is irrelevant to prion infections.

In terminal Creutzfeldt‐Jakob disease of humans, intraneuronal vacuoles gradually give rise an extensive sponge‐like state that occupies much of the cerebral cortex. These vacuoles may arise from lysosomes, whose function can be impaired by prion infections (Shim *et al*, 2016; Liberski, 2019). Lysosome maturation is controlled by the phosphoinositide diphosphate PI(3,5)P_2_ generated by the PIKfyve kinase. PIKfyve forms a complex with the phosphatase FIG4 and the scaffolding protein VAC14 on the cytosolic face of endosomes. Ablation of any of these proteins alters the PI(3,5)P_2_ levels, resulting in enlarged endolysosomes reminiscent of prion‐induced spongiosis (Chow *et al*, 2007; Zhang *et al*, 2007; Zolov *et al*, 2012). Here, we examine the role of PIKfyve and PI(3,5)P_2_ in prion‐induced spongiosis.

## Results

**Figure 1:**
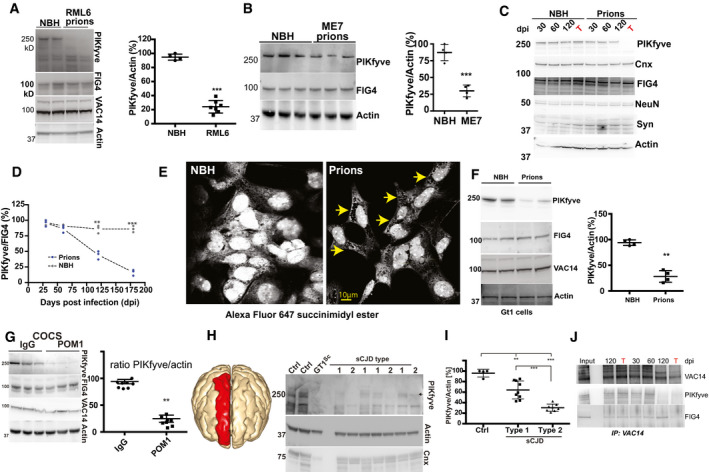
PIKfyve is universally depleted in prion diseases AConspicuous downregulation of PIKfyve, but not FIG4 or VAC14, in brain lysates of terminally sick mice intracerebrally inoculated with prions (RML). Control: Mice inoculated with non‐infectious brain homogenate (NBH). Right: Quantification of immunoblot. Each dot represents one mouse. ***P < 0.001; unpaired *t*‐test. Error bars represent s.e.m.BPIKfyve is selectively downregulated in mice infected with ME7 prions. Right: Quantification. Each dot represents one individual (*n* = 4/group). ***P < 0.001; unpaired *t*‐test. Error bars represent s.e.m.CTime course of PIKfyve downregulation in brains of prion‐infected mice (three mice/time point). PIKfyve (but not FIG4, Calnexin, NeuN, and synaptophysin) was suppressed between 60 and 120 dpi. T: terminal disease stage.DThe PIKfyve/FIG4 ratio gradually decreased in prion‐infected mice (unpaired *t*‐test). **P < 0.01; ***P < 0.001. Each dot represents one mouse.EPrion‐infected Gt1 cells (75 dpi) stained with Alexa‐Fluor 647‐succinimidyl ester which stains vacuoles negatively (arrows).FLoss of PIKfyve, but not FIG4 and VAC14, in prion‐infected Gt1 cells. Right: Quantification of immunoblots. Each dot represents an independent experiment. **P < 0.01; unpaired *t*‐test. Error bars represent s.e.m.G
*tg*a*20* COCS were treated with pooled IgG or POM1 (72 h). POM1 suppressed PIKfyve but not FIG4 and VAC14. Right: Quantification of immunoblots. Each dot represents an independent experiment. **P < 0.01; ***P < 0.001; unpaired *t*‐test. Error bars represent s.e.m.HImmunoblots of human CJD brains (red: sampled region). Gt1^Sc^: prion‐infected Gt1 cells. Loading controls: actin and Calnexin (Cnx).IQuantification of immunoblots revealed moderate and substantial PIKfyve loss in type 1 and type 2 CJD, respectively (unpaired *t*‐test). **P < 0.01; ***P < 0.001. Error bars represent s.e.m.JBrain homogenates from panel C were immunoprecipitated with anti‐VAC14 antibodies. FIG4 and PIKfyve did not co‐precipitate in prion‐infected brains at terminal disease.
Source data are available online for this figure.

Six C57BL/6 wild‐type mice were inoculated intracerebrally with RML scrapie prions (Rocky Mountain Laboratory strain, passage 6, originally derived from scrapie‐infected sheep) and sacrificed at terminal disease 184 ± 16 days post‐infection (dpi). For control, we inoculated four mice with non‐infectious brain homogenate (NBH, 190 dpi). Prion‐infected brains showed a profound reduction in PIKfyve, but not of FIG4 and VAC14 (Fig 1A). PIKfyve was not affected in other organs (Appendix Fig S1A). PIKfyve levels were also reduced in brains of mice infected with the ME7 strain of prions (Fig 1B) and in RML‐infected PrP^C^‐overexpressing *tg*a*20* mice (Fischer *et al*, 1996) (Appendix Fig S1B). Tumor susceptibility gene 101 (Tsg101), superoxide dismutase‐2 and Mahogunin‐1, which have been implicated in vacuolating brain diseases including genetic spongiform encephalopathies (He *et al*, 2003b; Doyotte *et al*, 2005; Izuo *et al*, 2015), were unchanged (Appendix Fig S1C). PIKfyve levels were decreased at 120 dpi and were profoundly depleted at the terminal stage (Fig 1C and D). PIKfyve is highly expressed in neurons (Zeisel *et al*, 2018), yet the neuronal proteins NeuN and synaptophysin were unaffected (Fig 1C), suggesting that its loss did not reflect the neuronal loss in scrapie‐sick mice.

We then infected murine Gt1 cells (Mellon *et al*, 1990) with RML prions. Gt1 cells have previously been reported to show appearance of vacuoles upon prion infections. At 75 dpi, we observed the appearance of cytoplasmic vacuoles accompanied by a substantial reduction in PIKfyve, but not of FIG4 or VAC14 (Fig 1E and F). PrP depletion from prion‐infected Gt1 cells by RNAi at 70 dpi largely suppressed cytoplasmic vacuolation (Appendix Fig S1D). Next, we administered RML prions to cerebellar organotypic cultured slices (COCS) from 9‐day‐old *tg*a*20* mice (Falsig & Aguzzi, 2008). At 45 dpi, we observed neurodegeneration accompanied by conspicuous PIKfyve depletion (Appendix Fig S1E and F). Finally, we exposed COCS for 72 h to the antibody POM1 which induces PrP^C^‐dependent neurotoxicity (Sonati *et al*, 2013) similarly to prion infections (Herrmann *et al*, 2015b). Again, this treatment depleted PIKfyve from COCS (Fig 1G).

We then asked if PIKfyve is lost in human sporadic Creutzfeldt‐Jakob disease (sCJD). Differential proteolysis of the misfolded prion protein (PrP^Sc^) discriminates two types of sCJD with distinct clinical courses and histopathological characteristics (Gambetti *et al*, 2003). We investigated 9 and 8 frontal cortex samples (Appendix Fig S1G and H) from patients who succumbed from type 1 and type 2 sCJD, respectively, as well as 4 non‐CJD controls, collected between 2002 and 2010 (Table EV1). PIKfyve levels were reduced in all cases, but the reduction was more profound in type 2 sCJD samples (Fig 1H and I) which also showed more extensive vacuolation (Appendix Fig S1H). Along with the earlier onset of disease in type 2 CJD (average: 57 vs. 63 years) (Iwasaki, 2017), this suggests that PIKfyve depletion may be a determinant of toxicity. Therefore, PIKfyve depletion is a feature of all investigated instances of prion infections.

We then studied the consequences of prion infection onto the composition of the PIKfyve tripartite complex. While VAC14 and FIG4 levels were not affected at any time point during the progression of prion disease, co‐immunoprecipitation experiments revealed that VAC14 lost its association with FIG4 and PIKfyve in prion‐infected brains at the terminal stage of the disease (Fig 1J and Appendix Fig S1O). Conversely, the siRNA knockdown of FIG4 or VAC14 in Gt1 cells using RNAi caused a significant PIKfyve reduction. The knockdown of FIG4 reduced VAC14 levels whereas the VAC14 knockdown did not alter FIG4 (Appendix Fig S1I). Hence, the prion‐induced PIKfyve depletion destabilizes the association between FIG4 and VAC14 without reducing their concentration.

Next, we investigated the mechanism of PIKfyve suppression by prions. Prion‐infected mice (Appendix Fig S1J), as well as prion‐infected or POM1‐treated COCS (Appendix Fig S1K and L, respectively), showed no alterations in PIKfyve, FIG4, and VAC14 brain mRNA levels. The relative expression of PIKfyve mRNA splice variants was similar between RML‐infected and control mouse brains (Appendix Fig S1M and N), and exploration of a splice alteration database during the progression of prion disease (Sorce *et al*, 2020) did not identify any differential expression of PIKfyve isoforms. Hence, PIKfyve suppression occurs post‐transcriptionally.

**Figure 2:**
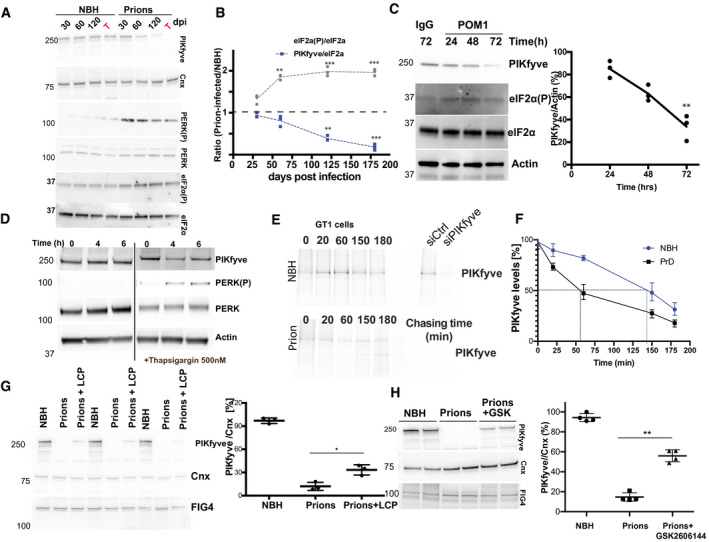
ER stress regulates PIKfyve expression levels ATime course of eIF2α and PERK phosphorylation in prion‐inoculated C57BL/6 mice. T: terminal disease.BPIKfyve/eIF2α and eIF2α(P)/eIF2α ratios assessed from Western blots as in A (*n* = 3/group/time point). PIKfyve levels gradually decreased after 60 dpi whereas eIF2α became phosphorylated from 30 dpi until terminal disease. eIF2α phosphorylation and pPIKfyve depletion in POM1‐treated *tg*a*20* COCS. **P < 0.01; ***P < 0.001; unpaired *t*‐test; error bars represent s.e.m.CTreatment of COCS with POM1 reduces the levels of PIKfyve starting 48 h post‐treatment. Each dot represents an individual experiment. Statistics: Unpaired *t*‐test. **P < 0.01.DTransient PIKfyve suppression in thapsigargin‐treated Gt1 cells with recovery after 6 h. PERK phosphorylation confirmed ER stress.EGt1 cells were inoculated with NBH or prions (60 dpi), pulsed with [^35^S]Met/Cys (20 min, 37°C), and immediately lysed or chased without ^35^S (37°C, ≤3 h). For control, cells were treated with scrambled siRNA or siRNA against PIKfyve. Proteins were immunoprecipitated with antibodies to PIKfyve and subjected to SDS–PAGE and autoradiography (*n* = 3 individual biological replicates).FAutoradiographic signals expressed as percentage of the initial PIKfyve levels. Prion infection shortened the half‐life of PIKfyve from 144 to 52 min. First‐order kinetics was assumed. Error bars represent s.e.m and were calculated based on three individual biological replicates.GLeft: partially restored PIKfyve levels in prion‐infected *tg*a*20* mice treated with LIN5044 (LCP) or vehicle. Right: quantification of the Western blot. Each dot represents an individual biological replicate. *P: 0.05; unpaired *t*‐test. Error bars represent s.e.m.HLeft, *tg*a*20* COCS were inoculated with RML or NBH, optionally treated with GSK2606414 starting at 21 dpi, and lysed at 35 dpi. Depletion of PIKfyve in prion‐infected samples was largely rescued by GSK2606414 (GSK). Right, quantification of Western blot. Each dot represents an individual biological replicate. Statistics: Unpaired *t*‐test. **P < 0.01; unpaired *t*‐test; error bars represent s.e.m.
Source data are available online for this figure.

Prion diseases induce a chronic unfolded protein response (UPR) in the endoplasmic reticulum (ER) leading to eIF2α phosphorylation and translational suppression (Moreno *et al*, 2012), and UPR inhibitors can mitigate spongiosis (Moreno *et al*, 2013). We first compared the evolution of the UPR and of PIKfyve depletion in various prion models. In prion‐infected mouse brains, eIF2α phosphorylation was detectable at 30 dpi, whereas PIKfyve decreased gradually starting at 60 dpi (Fig 2A and B). In COCS, eIF2α became phosphorylated 24 h after exposure to POM1, yet PIKfyve was depleted only after 72 h (Fig 2C). Hence, a chronic UPR precedes downregulation of PIKfyve *in vivo* and *ex vivo*. We then induced an acute UPR in Gt1 cells with thapsigargin (0.5 µM), an inhibitor of endoplasmic reticulum Ca2 ATPase, resulting in transient but substantial PIKfyve depletion (Fig 2D and Appendix Fig S2A and B). This did not result in generation of vacuoles (S2C). We next assessed PIKfyve translation during the UPR and in prion‐infected cells. Naïve, prion‐infected (75 dpi), or thapsigargin‐treated GT1 cells (4 h) were labeled with [^35^S]methionine/cysteine for 40 min, and PIKfyve was immunoprecipitated. Translation of PIKfyve was not altered in prion infection or UPR (S2D).

We then asked if the half‐life of PIKfyve is altered in prion infection. Naïve or prion‐infected Gt1 cells (60 dpi) were exposed to a [^35^S]methionine/cysteine pulse (20 min) followed by chasing in ^35^S‐free culture medium. Prion infection reduced the half‐life of immunopurified [^35^S]PIKfyve from 144 ± 14 to 54 ± 12 min (Fig 2E and F).

If PIKfyve depletion mediates prion neurotoxicity, it may be attenuated by antiprion therapeutics. The conjugated polythiophene LIN5044 binds PrP^Sc^, antagonizes prion replication (Sigurdson *et al*, 2007; Margalith *et al*, 2012), delays prion pathogenesis *in vivo*, and reduces spongiosis (Herrmann *et al*, 2015a). Remarkably, prion‐infected mice treated with LIN5044 (LCP) not only showed prolonged survival but also partially restored PIKfyve levels (Fig 2G). We then administered GSK2606414, an inhibitor of the protein kinase RNA‐like endoplasmic reticulum kinase (PERK) arm of the UPR (Moreno *et al*, 2013), to prion‐infected *tg*a*20* COCS at 21 dpi (Appendix Fig S2E). GSK2606414 partially restored PIKfyve levels (Fig 2H). Similarly, ISRIB, a drug which renders cells insensitive to eIF2α phosphorylation, partially rescued PIKfyve levels in prion‐infected Gt1 cells (75 dpi) (Appendix Fig S2F). These data support the notion that chronic ER stress is a direct cause of PIKfyve downregulation.

**Figure 3:**
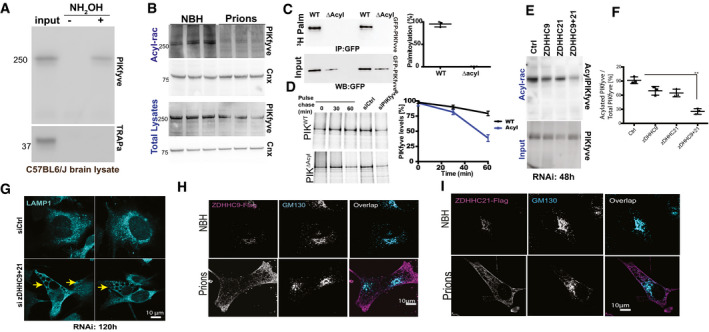
Prion infection causes deacylation and degradation of PIKfyve AAcyl‐rac captured hydroxylamine‐cleavable, acylate PIKfyve from mouse brains. Control: TRAPα, a non‐acylated protein. Input: 10% of lysate.BBrain lysates from prion‐inoculated mice (60 dpi) precipitated by Acyl‐rac. PIKfyve, but not Calnexin (Cnx), was deacylated in prion‐infected samples. Each lane represents an individual mouse. Lower panel: lysate used for Acyl‐rac.CAutoradiography of Gt1 cells transfected with PIKfyve‐GFP or PIKfyve^Δacyl^ (24 h), metabolically labeled with 3H‐palmitate (2 h), and immunoprecipitated with anti‐GFP antibodies. Lower panel: Western blot on immunoprecipitates using anti‐GFP antibodies. Error bars represent s.e.m. (*n* = 3 individual biological replicates).DGt1 cells transiently expressing PIKfyve‐GFP or PIKfyve^ΔAcyl^ (24 h) were subjected to a [^35^S]Met/Cys pulse (20 min, 37°C) and immediately lysed or chased (60 min). Proteins were immunoprecipitated with anti‐GFP antibody and subjected to SDS–PAGE and autoradiography. Lysates from cells treated with siRNA against PIKfyve were also pulsed and immunoprecipitated and subjected to autoradiography to ensure correct band is detected. Error bars represent s.e.m. (*n* = 3 individual biological replicates).E, FAcyl‐rac of Gt1 cells subjected to siRNA against zDHHC9, zDHHC21, or both (72 h post‐transfection), showing synergistic decrease of PIKfyve acylation by suppression of zDHHC9 and 21 (quantified in F). Each dot represents a separate experiment (unpaired *t*‐test). **P < 0.01. Error bars represent s.e.m.GGt1 cells were transfected with siRNA against zDHHC9+21 and stained with LAMP1. Arrows: vacuoles.H, IFlag‐tagged zDHHC9 or zDHHC21 expressed in Gt1 cells were transfected with plasmids encoding. After 72 h, zDHHC9/21 (magenta) were localized to the Golgi apparatus (GM130^+^, cyan) in NBH‐exposed cells but not in prion‐infected cells. *n* = 3 individual biological replicates.
Source data are available online for this figure.

Dynamic, reversible acylation controls protein stability (Gao & Hannoush, 2018) and can be affected by the UPR (Lynes *et al*, 2013). Being juxtaposed to endosomal membranes, PIKfyve is a plausible candidate for acylation. Acyl‐resin assisted capture (acyl‐rac) of mouse brain homogenates showed that PIKfvye, but not the cysteine‐less translocon‐associated protein‐α (TRAPα), was acylated (Fig 3A). Prion‐infected samples, however, showed massively reduced PIKfyve acylation already at 60 dpi, a time point showing vigorous UPR but only minimal depletion of total PIKfyve (Figs 2A and 3B, and Appendix Fig S3A).

An acylation prediction database (Blanc *et al*, 2015) pointed to cysteine residues 202 and 203 as potential palmitoylation sites in all three isoforms of PIKfyve (Appendix Fig S3B). We therefore expressed PIKfyve‐GFP or PIKfyve^C202A;C203A^‐GFP (henceforth PIKfyve^Δacyl^), in which both residues were replaced by alanines, in Gt1 cells. Indeed, acyl‐rac showed that mutagenesis of cysteine residues 202 and 203 prevented PIKfyve acylation (Appendix Fig S3C). To corroborate this finding, we radiolabeled cells expressing PIKfyve‐GFP or PIKfyve^Δacyl^ with ^3^H‐palmitate (2 h). Immunoprecipitation of PIKfyve using anti‐GFP antibodies from the radiolabeled lysates revealed that PIKfyve‐GFP, but not PIKfyve^Δacyl^, underwent acylation (Fig 3C). We then assessed if loss of acylation leads to destabilization of PIKfyve. GT1 cells expressing either PIKfyve‐GFP or PIKfyve^Δacyl^ were pulsed with a [^35^S]methionine/cysteine pulse (20 min) followed by chase in radiolabel‐free medium for 60 min. At 60 min, PIKfyve^Δacyl^ showed considerable decay, suggesting its destabilization and degradation (Fig 3D). We next sought to understand whether loss of acylation would mislocalize PIKfyve, which normally resides in late endosomes and lysosomes. The expression of either PIKfyve‐GFP or PIKfyve^Δacyl^ resulted in a punctate GFP signal throughout Gt1 cells and in extensive vacuolation (Appendix Fig S3D).

We next investigated the function of the acylation machinery in prion infections. The mouse genome encodes 23 acyltransferases characterized by a zinc finger domain and an aspartate‐histidine‐histidine‐cysteine tetrapeptide (zDHHC), denoted zDHHC1–24. Quantitative real‐time PCR (qPCR) on terminally scrapie‐sick mouse brains failed to identify altered mRNA levels of any zDHHC member (Appendix Fig S3E). We then mined a database of transcriptional changes during the course of prion infection (Sorce *et al*, 2020). None of the zDHHC mRNAs were altered at any stage of the disease (Appendix Fig S3F). Next, we suppressed each zDHHC individually by siRNA. All zDHHC mRNAs were reduced by > 80%. After 72 h, cells were harvested and subjected to acyl‐rac. Only the knockdown of zDHHC9 and zDHHC21 modestly reduced the acylation of PIKfyve (Fig 3E and Appendix Fig S3G). We next knocked down zDHHC9 and 21 individually vs. simultaneously in Gt1 cells. Acyl‐rac at 48 h showed synergy of the zDHHC9/21 siRNA mix (Fig 3E and F). Crucially, at 120 h the zDHHC9/21 siRNA mix induced not only a downregulation of PIKfyve but also the appearance of vacuoles (Appendix Fig S3H and Fig 3G) similar to those of prion‐infected cells.

Next, we expressed in Gt1 cells zDHHC9 and zDHHC21 fused to a C‐terminal flag‐tag. While zDHHC9 and zDHHC21 localized to Golgi complexes of NBH‐treated cells, in prion‐infected cells both enzymes became drastically mislocalized to the cell periphery (Fig 3H and I and Appendix Fig S3I). The mislocalization of zDHHC9/21 may explain the loss of PIKfyve acylation and its degradation, thereby driving spongiogenesis. Finally, we investigated whether triggering an acute UPR would suffice to delocalize zDHHC9/21. However, thapsigargin‐treated (4 h) cells showed orthotopical localization of zDHHC9 and 21 in the Golgi, indicating that acute UPR induction does not suffice to mislocalize them (Appendix Fig S3J).

### Cellular consequences of prion‐induced spongiosis

**Figure 4:**
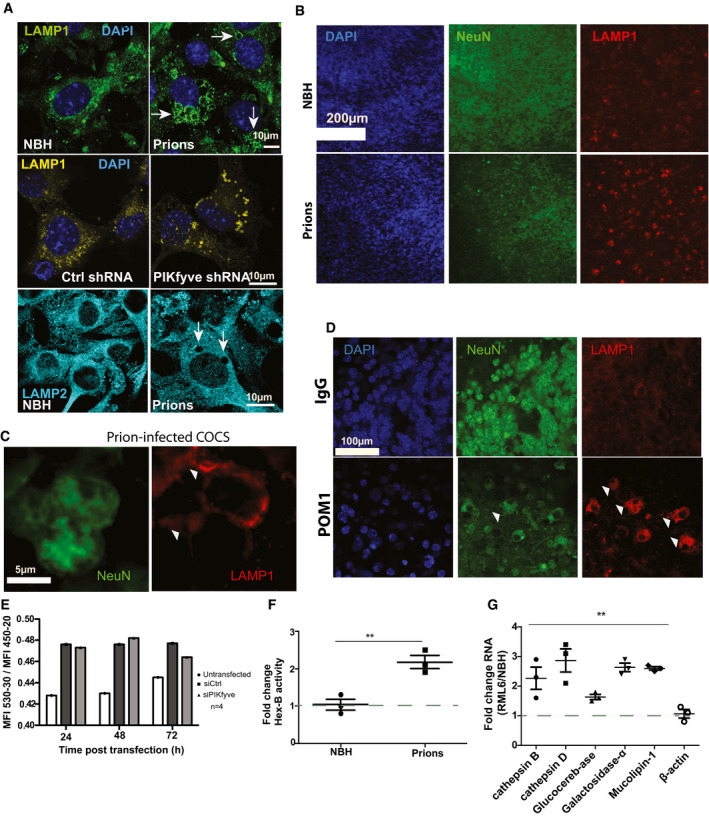
Loss of PIKfyve induces lysosomal defects AUpper row: prion‐infected Gt1 cells (75 dpi) immunostained for LAMP1 showing prominent vacuoles. Control: NBH‐inoculated cells. Middle row: Gt1 cells transfected with shRNA targeting PIKfyve or luciferase (control) for 5 days and immunostained for LAMP1. Depletion of PIKfyve resulted in LAMP1^+^ vacuoles (yellow). DAPI: nuclear stain. Lower row: prion‐infected and NBH‐treated cells (75 dpi) contained LAMP2^‐^ vacuoles. *n* = 3 individual biological replicates.BPrion‐infected *tg*a*20* COCS (45 dpi) immunostained with NeuN and LAMP1. Control: NBH‐exposed COCS. Nuclei: DAPI. Prion infection induced LAMP1 upregulation in neurons (quantification: Appendix Fig S4A; *n* = 3 individual biological replicates).CHigher magnification showing LAMP1^+^ vacuoles (arrowheads) in prion‐infected COCS.D
*tg*a*20* COCS treated with POM1 or control IgG (10 days) and immunostained with NeuN and LAMP1. Nuclei: DAPI. POM1‐treated COCS showed increased LAMP1 expression (quantification: Appendix Fig S4B). NeuN^+^ cells exhibited vacuoles (arrowheads).EGt1 cells were transfected with control siRNA (scrambled) or siRNA targeting PIKfyve for up to 72 h and stained with LysoSensor Yellow/Blue. Cells were gated based on emission spectra, and mean fluorescence intensities (MFIs) per sample were quantified. Increased 530/450 MFI ratio indicates enrichment of acidic compartments. No acidic compartment expansion in PIKfyve‐depleted cells. *n* = 4 individual biological replicates. Error bars represent s.e.m.Fβ‐Hexosaminidase A activity in brain lysates from terminally scrapie‐sick and NBH‐inoculated mice. Control: NBH‐inoculated mice. Prion‐infected brain lysates showed 2‐ to 2.5‐fold increased activity. Panels show independent triplicates. Statistics: unpaired *t*‐test. **P < 0.01. Error bars represent s.e.m.GLysosomal enzymes, but not β‐actin, were elevated in brains of terminally scrapie‐sick mice. Panels show independent triplicates. Statistics: ANOVA. **P < 0.01. Error bars represent s.e.m.

Since PIKfyve is necessary for the maturation of late endosomes into lysosomes, prion‐associated spongiosis may result from a blockade of endosomal maturation. We found that LAMP1, a marker for late endosomes/lysosomes, was associated with vacuoles in both prion‐infected Gt1 cells and in Gt1 cells depleted of PIKfyve using shRNA (Fig 4A). LAMP2, a lysosome marker, did not associate with the vacuoles (Fig 4A). We wondered whether the association of LAMP1 with vacuoles can also be observed in other model systems of prion disease. We infected COCS generated from 9‐day‐old *tg*a*20* mice with RML. At 50 dpi, we detected LAMP1 upregulation in neurons (Fig 4B and Appendix Fig S4A). As in Gt1 cells, vacuoles in COCS were lined by LAMP1 (Fig 4C). Treatment of *tg*a*20* COCS with POM1 (10 days) also resulted in microvacuolation and LAMP1 upregulation in neurons (Fig 4D and Appendix Fig S4B). Electron microscopy revealed that POM1‐induced microvacuoles had a limiting membrane and were devoid of any electron‐dense material, similarly to prion‐induced vacuoles (Appendix Fig S4C). Furthermore, vacuoles of prion‐infected Gt1 cells were lined with the endosomal marker SARA (Appendix Fig S4D). These results suggest that spongiosis originates from late endosomal/lysosomal compartments. To differentiate between these two possibilities, we stained PIKfyve‐depleted Gt1 cells (72 h after siRNA transfection) with the ratiometric pH probe LysoSensor Yellow/Blue (5 µM, 5 min). Flow cytometry revealed no enhanced acidification in vacuolated cells (Fig 4E), suggesting that vacuoles represent stalled prelysosomal compartments.

Increased activity of LAMP1 and other lysosomal enzymes is a frequent feature of lysosomal dysfunction. Indeed, we found a threefold increase in the enzymatic activity of lysosomal hexosaminidase‐β in brains of prion‐infected mice (Fig 4F). In addition, we monitored the RNA expression levels of cathepsin‐D, cathepsin‐A, glucocerebrosidase, α‐galactosidase, and mucolipin‐1, previously shown to be upregulated in lysosomal diseases (Sardiello *et al*, 2009), and found all of them to be upregulated in brains of terminally scrapie‐sick mice (Fig 4G).

Lysosomes undergo cycles of fission and fusion, which are impaired by the absence of PIKfyve, resulting in coalesced endolysosomes (Choy *et al*, 2018). The consequences include nuclear translocation of TFEB and depletion of TRPML1, a channel controlling lysosomal size (Cao *et al*, 2017; Zhong *et al*, 2017; Di Paola *et al*, 2018). TRPML1, whose lysosomal association relies on PIKfyve (Isobe *et al*, 2019), showed altered localization in prion‐infected Gt1 cells (Appendix Fig S4E). We next investigated the fidelity of endocytosis by pulsing prion‐infected Gt1 cells (75 dpi) with Alexa‐488 transferrin for 10 min on ice, followed by washing and chasing for 30 min at 37°C. Transferrin was detected in lysosomes of both prion‐infected and NBH‐treated cells (Appendix Fig S5A and B). These results suggest that endocytosis is not globally affected in prion‐infected cells.

**Figure 5:**
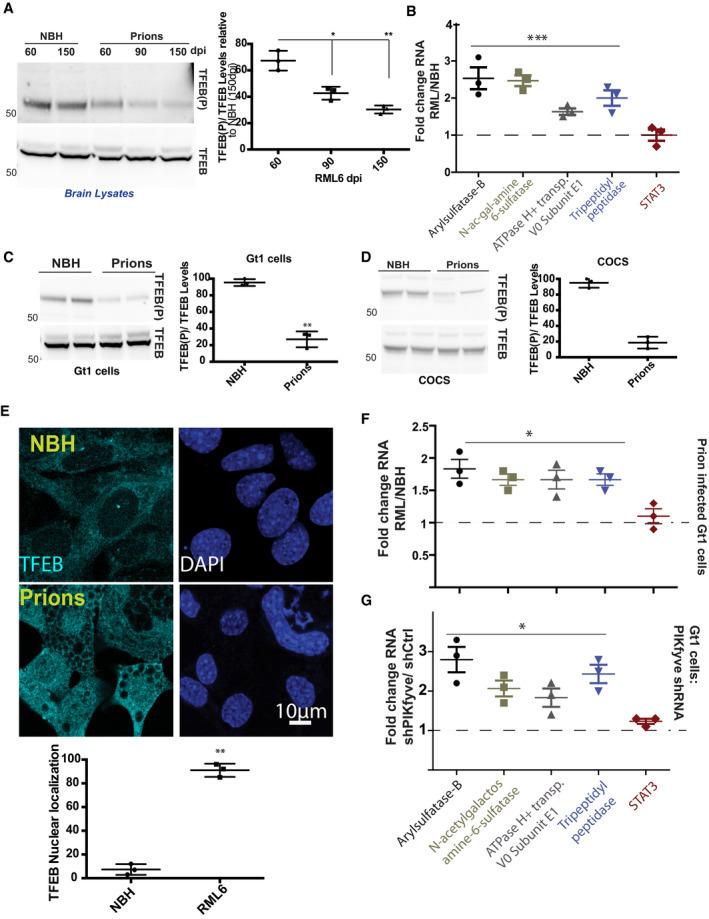
Prions induce lysosomal dysregulation via TFEB ATime course of TFEB phosphorylation in prion‐infected C57BL/6 mice. Total TFEB was unchanged but reduced TFEB phosphorylation was evident at ≥ 90 days. Quantification: Each dot represents an individual biological replicate. Statistics: Unpaired *t*‐test. *P: 0.05; **P < 0.01. Error bars represent s.e.m.BTFEB‐controlled transcripts in brains of terminally scrapie‐sick C57BL/6 mice were measured by qPCR. Prion infection led to upregulation of these genes, but not of STAT3. Control: NBH‐inoculated mice. ANOVA on independent triplicates. ***P < 0.001. Error bars represent s.e.m.CPrion‐infected Gt1 cells (75 dpi) showed reduced TFEB phosphorylation. Total levels of TFEB remain unchanged. Quantification: Each dot represents an individual biological replicate. Statistics: Unpaired *t*‐test. **P < 0.01. Error bars represent s.e.m.DCOCS prepared from *tg*a*20* mice were infected with RML prions and cultured for 5 weeks. Cell lysates were prepared at 4 wpi followed by Western blot analysis using anti‐phospho‐TFEB antibody. Activated (dephosphorylated) TFEB was increased in RML‐infected COCS. Total levels of TFEB remain unchanged. Quantification: Each dot represents an individual biological replicate. Statistics: Unpaired *t*‐test. Error bars represent s.e.m.EGt1 cells (as in D) were fixed and stained with anti‐TFEB antibody. Prion‐infected cells showed nuclear translocation of TFEB. DAPI: Blue. Quantification: Each dot represents an individual biological replicate (30 images were quantified per replicate). Statistics: Unpaired *t*‐test. **P < 0.01. Error bars represent s.e.m.FTFEB‐responsive genes were upregulated in Gt1 cells chronically infected with RML prions (75 dpi). Panels depict independent triplicates. Statistics: ANOVA. *P: 0.05. Error bars represent s.e.m.GTFEB‐responsive genes were upregulated in cells transfected with shPIKfyve for 5 days compared with shCtrl (shRNA against luciferase). Panels depict independent triplicates. Statistics: ANOVA. *P: 0.05. Error bars represent s.e.m.
Source data are available online for this figure.

Upon dephosphorylation, transcription factor EB (TFEB) translocates to the nucleus and promotes coordinated expression of lysosomal genes (Sardiello *et al*, 2009; Medina *et al*, 2015; Xiao *et al*, 2015). TFEB became progressively dephosphorylated in prion‐infected mouse brains whereas total TFEB levels remained constant (Fig 5A). We then examined a subset of TFEB‐regulated lysosomal genes in terminally prion‐sick brains. The mRNA levels of all investigated genes were conspicuously increased, whereas the non‐lysosomal gene STAT3 was not (Fig 5B). These findings could reflect the vivacious proliferation of activated microglia in terminal prion disease. We therefore investigated the lysosomal status of Gt1 cells (Fig 5C) and COCS (Fig 5D). We found that prion infection led to TFEB dephosphorylation in both models. Moreover, prion‐infected Gt1 cells (75 dpi) displayed nuclear translocation of TFEB (Fig 5E) and prion‐infected Gt1 cells (75 dpi), as well as Gt1 cells depleted of PIKfyve by shRNA, showed upregulation of TFEB‐responsive genes (Fig 5F and G). This disproves that lysosomal gene upregulation merely indicates distorted tissue composition and suggests that TFEB‐mediated reactions are a cell‐autonomous consequence of PIKfyve depletion. Finally, we tested the relationship between TFEB and lysosomal homeostasis by treating prion‐infected Gt1 cells with siRNA against TFEB. 72 h post‐siRNA transfection, the expression of TFEB‐responsive genes was normalized, yet the vacuolation of prion‐infected cells was unaltered (Appendix Fig S5C–E), suggesting that TFEB‐related lysosome pathologies may be a consequence rather than a cause of spongiosis.

**Figure 6:**
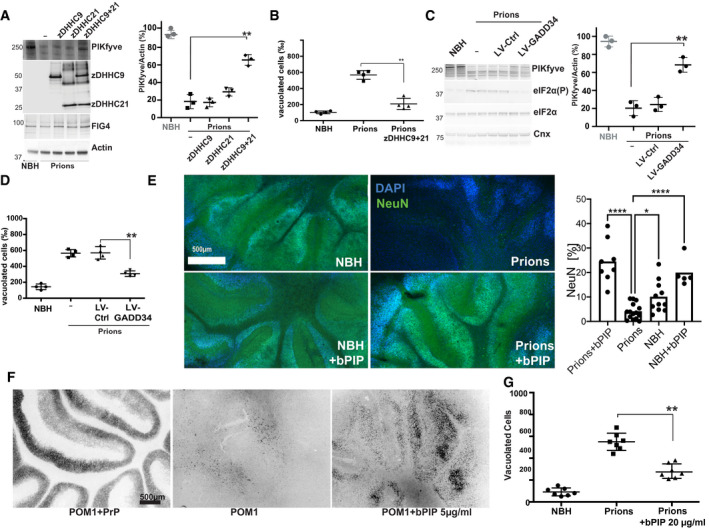
PI(3,5)P_2_ attenuates spongiosis and lysosomal dysfunction ATransient overexpression of Flag‐tagged zDHHC9 + 21 (72 h) restored PIKfyve levels in prion‐infected Gt1 cells. Right: Western blot quantification. Each dot represents an individual experiment (unpaired *t*‐test). **P < 0.01. Error bars represent s.e.m.BCotransfection of zDHHC9 + 21 reduced vacuolation in prion‐infected Gt1 cells. Each dot represents a separate experiment (1,000 cells/experiment, χ^2^: *P* < 0.001). **P < 0.01; unpaired *t*‐test. Error bars represent s.e.m.CPrion‐infected Gt1 cells were lentivirally transduced with GADD34 at 75 dpi. Control: empty lentiviral vector. At 79 dpi, GADD34 overexpression had normalized eIF2α phosphorylation and restored PIKfyve levels. Right: Quantification of Western blots (*n* = 3; unpaired *t*‐test). Each dot represents an individual experiment. **P < 0.01. Error bars represent s.e.m.DNumber of vacuolated cells from the experiment shown in C. GADD34 expression rescued the total number of vacuolated cells (1,000 cells/experiment, χ^2^: *P* < 0.001). Each dot represents an individual experiment. **P < 0.01. Error bars represent s.e.m.E
*tg*a*20* COCS were infected with RML and optionally treated with bPIP (5 µg/ml). At 45 dpi, NeuN morphometry revealed ablation of cerebellar granule layer (CGN) in prion‐infected slices and its rescue by bPIP. Control: NBH‐treated COCS. Each dot represents an individual slice (Statistics: ANOVA). *P < 0.05. ****P < 0.0001.F
*tg*a*20* COCS were treated with POM1 (optionally pre‐blocked with recPrP) and treated with bPIP (5 µg/ml). At 14 dpi, NeuN morphometry revealed POM1‐induced ablation of cerebellar granule layer (CGL) and rescue by bPIP.GPrion‐infected Gt1 cells were treated with bPIP for 3 days. The number of vacuolated cells was reduced (1,000 cells/experiment, χ^2^: *P* < 0.001); Statistics: Chi‐square test. Each dot represents an individual experiment. **P < 0.01. Error bars represent s.e.m.
Source data are available online for this figure.

The following hierarchy of events emerges from these findings. Chronic activation of the UPR leads to mislocalization of acyltransferases. This, in turn, compromises PIKfyve acylation, reduces its half‐life, and destabilizes its association with VAC14 and FIG4. The resulting reduction in PI(3,5)P_2_ stalls lysosome maturation and induces inappropriate TFEB‐dependent transcriptional responses. We challenged this model by interfering with each of its predicted checkpoints. Firstly, we asked whether overexpression of zDHHC9 and zDHHC21 might restore PIKfyve levels and abrogate vacuole generation in prion‐infected Gt1 cells (75 dpi). Indeed, transfection (72 h) with both zDHHC9 and zDHHC21 partially restored PIKfyve levels and reduced the number of vacuolated cells (Fig 6A and B and Appendix Fig S6A). Secondly, we attenuated the UPR in prion‐infected Gt1 cells (75 dpi) by lentiviral transduction of GADD34 which dephosphorylates eIF2α (Rojas *et al*, 2015). Again, we found restoration of PIKfyve levels and a significant decrease in the frequency of vacuolated cells (Fig 6C and D). Besides confirming that increasing PIKfyve levels suffices to prevent vacuolation, these results establish the directionality of chronic UPR, delocalization of acyltransferases and PIKfyve depletion. Thirdly, we reasoned that prion‐induced spongiosis could be repressed by the PIKfyve adduct, PI(3,5)P_2_. We treated prion‐infected *tg*a*20* COCS for 45 days with bodipy‐PI(3,5)P_2_ (bPIP), a fluorescent water‐soluble analog of PI(3,5)P_2_ (5 µg/ml). Infection with RML led to complete ablation of cerebellar granule layer (CGN), which was attenuated by bPIP (Fig 6E). This effect was specific to phosphorylation at carbons 3 and 5, as bodipy‐PI(4,5)P2 failed to prevent the ablation of CGN in prion‐infected COCS (Appendix Fig S6B). Furthermore, bPIP reduced the occurrence of LAMP1^+^ vacuoles in prion‐infected COCS and restored the homeostasis of TFEB‐responsive genes (Appendix Fig S6C and D). Next, we treated *tg*a*20* COCS with POM1 for 14 days in the presence or absence of bPIP. The subsequent ablation of the CGN was again attenuated by bPIP (Fig 6F). We then treated prion‐infected Gt1 cells with bPIP (70 dpi, 20 µg/ml). After 12 h, > 60% of cells incorporated bPIP into lysosomes (Appendix Fig S6E and F). Fresh bPIP, or water for control, was added every 12 h over 72 h (Appendix Fig S6G). bPIP reduced the number of vacuolated cells (Fig 6G) and restored the homeostatic expression of TFEB‐responsive genes (Appendix Fig S7A), but did not alter the accumulation of PrP^Sc^ (Appendix Fig S7B). Hence, bPIP acts downstream of PrP^Sc^ generation. To further probe the specificity of bPIP, we depleted PIKfyve from Gt1 cells using shRNA. The ensuing vacuolation was conspicuously reduced by bPIP (Appendix Fig S7C).

## Discussion

Through multiple lines of evidence, we show that PIKfyve depletion is a universal feature of prion pathology. It occurs in human prion diseases, in mice infected with distinct strains of scrapie, in prion‐infected cell lines, and after administration of prion‐mimetic antibodies. It is conserved across species and experimental models, and the virulence of human prion diseases correlates with the extent of PIKfyve depletion. Furthermore, a wealth of observations points to PIKfyve as the crucial link between prion infection and neuronal spongiosis. Prion infections activate the PERK arm of the UPR (Moreno *et al*, 2013). PERK inhibitors prevented spongiosis and restored PIKfyve levels, whereas transient UPR induction favored PIKfyve degradation. Hence, a chronic neuronal UPR occurs upstream of PIKfyve suppression.

But what causes PIKfyve disappearance? We found that PIKfyve is acylated by the two acyltransferases, zDHHC9/21, which are highly expressed in the brain and are associated with neurodegenerative diseases (Fukata & Fukata, 2010; Young *et al*, 2012; Hornemann, 2015; Cho & Park, 2016). In addition to tethering proteins to membranes, acylation regulates their stability by preventing ubiquitylation and degradation (Linder & Deschenes, 2007; Gao & Hannoush, 2018). Conversely, deacylation may destabilize protein complexes and is implicated in Alzheimer’s disease, Huntington’s disease, and schizophrenia. Both prion infection and prion‐mimetic antibodies induced PIKfyve deacylation, and its kinetics suggests that the UPR modulates the acylation machinery. The localization of zDHHC21 at the Golgi/plasma membrane (Pedram *et al*, 2012) was altered by prion infection, suggesting a role in PIKfyve destabilization and degradation.

### Molecular basis for vacuole generation

The spongiosis and tubulovesicular bodies of prion diseases resemble lysosomes and multivesicular bodies (Laszlo *et al*, 1992). Lysosomes exchange their contents through fusion and fission, whose impairment by PIKfyve deficiency forces the coalescence of endolysosomes into large vacuoles (Choy *et al*, 2018). The PIKfyve adduct PI(3,5)P_2_, a crucial regulator of endolysosomal homeostasis (Ikonomov *et al*, 2009; McCartney *et al*, 2014; Min *et al*, 2014), suppressed vacuolation in prion‐infected cells. We therefore posit that the PIKfyve‐related deficiency of PI(3,5)P_2_ represents the proximal cause of spongiosis. We did not find any evidence for the controversial suggestion that Mahogunin participates in prion‐induced vacuolation (Chakrabarti & Hegde, 2009; Gunn & Carlson, 2013; Silvius *et al*, 2013). Neuronal vacuolation may cause neuronal dysfunction in multiple ways beyond the mechanical hindrance of cytosolic functions. After both prion infection and PIKfyve ablation, we observed nuclear translocation of TFEB and delocalization of TRPML1, a channel controlling lysosomal size (Cao *et al*, 2017; Zhong *et al*, 2017; Di Paola *et al*, 2018). Both TFEB knockdown and PI(3,5)P_2_ administration prevented the upregulation of lysosomal target genes, but the former did not counteract spongiosis indicating that TFEB activation is a consequence rather than a cause of spongiogenesis.

**Figure 7:**
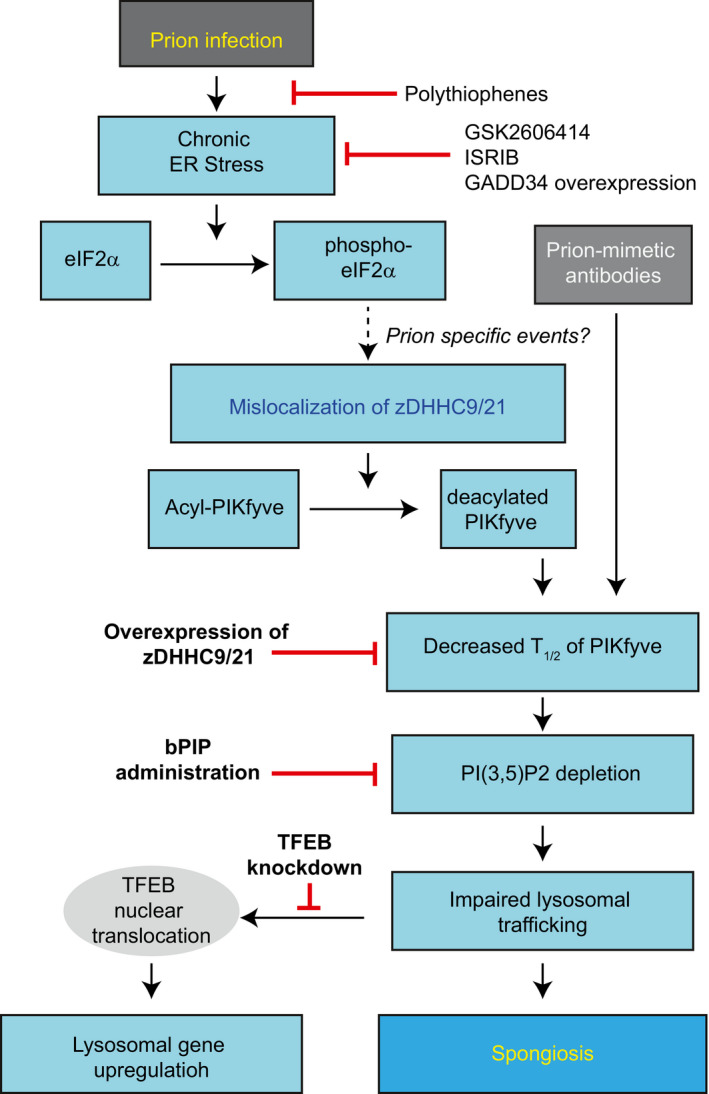
A comprehensive model of spongiogenesis Prion infection result a cascade of events affecting a number of enzymes in various cellular compartments, and eventually resulting in depletion of PI(3,5)P_2_ as the proximal cause of spongiosis. Red bars indicate therapeutic interventions.

The above observations point to a hierarchy of events that start with sustained UPR induction, eIF2α phosphorylation, and PERK activation. This leads to the delocalization of acyltransferases, resulting in impaired PIKfyve acylation and disruption of the ternary PIKfyve‐VAC14‐FIG4 complex. Deacylated PIKfyve then undergoes rapid ubiquitination and degradation, which causes PIP(3,5)_2_ levels to drop and endolysosomes to grow into vacuoles. Ultimately, these changes cause TFEB dephosphorylation and upregulation of lysosomal enzymes (Fig 7). The vesicular aberrations caused by PI(3,5)P_2_ deficiency impair synaptic functions (Seebohm *et al*, 2012), suggesting a direct impact onto the neuronal dysfunction characteristic of prion diseases.

### Therapeutic outlooks and caveats

The findings described here point to potential therapeutic targets. Blocking UPR or increasing the expression of zDHHC9 and 21 sufficed to rescue the PIKfyve levels and the number of vacuolated cells in the cell line model system of vacuolation. UPR inhibitors are being tested for their therapeutic potential (Hughes & Mallucci, 2019), and it will be interesting to see whether any of them can also rescue spongiosis in prion infections. Acyltransferase activity may also be targeted, particularly because PIKfyve is processed only by two cellular zDHHCs. Several UPR inhibitors are being tested for their therapeutic potential (Hughes & Mallucci, 2019), and it will be interesting to see whether any of them can also inhibit spongiogenesis in prion infections. The manipulation of zDHHCs could be an additional strategy against prion toxicity. Administration of PI(3,5)P_2_ analogues could also be explored, but it may be difficult to achieve effective pharmacokinetics. If these challenges can be overcome, the outlined approaches may provide an opportunity to target novel effectors of toxicity in prion diseases.

PIKfyve inhibition reduces the transport of α‐synuclein, and tau aggregates into lysosomes, preventing its dissemination into the cytosol (preprint: See *et al*, 2021; Soares *et al*, 2021), and was advocated as a therapeutic strategy for neurodegeneration. However, our results imply the existence of important caveats. The spongiosis caused by chronic PIKfyve depletion may offset any therapeutic gains and may even precipitate disastrous neurodegeneration.

Vacuoles can occur in additional UPR‐causing diseases (Wiersma *et al*, 2019), including granulo‐vacuolar degeneration in Alzheimer's disease (Funk & Kuret, 2012) and rimmed vacuoles in inclusion‐body myositis (Villanova *et al*, 1993). This begs the question whether PIKfyve may play a role in vacuolating diseases other than prion infections. However, no other neurodegenerative disease shows the extensive vacuolation characteristic of prion infections. Therefore, prion infections may trigger additional pathways which act in concert with the UPR to downregulate PIKfyve and/or to amplify the deleterious consequences thereof.

### Limitations and future directions

In the current study, we show that chronic ER stress and PIKfyve depletion link prion infections to the spongiform changes typical of prion diseases. However, chronic ER stress alone does not suffice to induce spongiosis, suggesting the existence of additional unknown prion‐specific events causing zDHHC9/21 delocalization and subsequent PIKfyve deacylation and destabilization. Moreover, while we found that restoring PI(3,5)P_2_ levels can rescue vacuolation in multiple paradigms, we do not fully understand the mechanism by which PIKfyve depletion affects lysosomal function. Possible strategies toward clarifying these issues may include unbiased forward genetic screens (e.g., using arrayed CRISPR libraries). When coupled to appropriate phenotypic assays (e.g., microscopy‐based high‐throughput analyses), such screens might have the power to identify the remaining components of the PIKfyve‐vacuolation‐neurotoxicity axis and, ultimately, to provide druggable therapeutic targets.

## Materials and Methods

Informed consent was obtained from all relevant subjects for all the experiments performed.

### Mice and Intracerebral prion inoculations

Mice were bred in high hygienic grade facilities and housed in groups of 3–5, under a 12‐h light/12‐h dark cycle (from 7 am to 7 pm) at 21 ± 1°C, with sterilized food (Kliba No. 3431, Provimi Kliba, Kaiseraugst, Switzerland) and water *ad libitum*. Animal care and experimental protocols were in accordance with the Swiss Animal Protection Law and approved by the Veterinary Office of the Canton of Zurich (permits 130/2008, 41/2012, 40/2015).

The following mice were used in the current study: C57BL/6, and *tg*a*20* (B6;129‐Tg(Prnp)a20Cwe Prnp<tm1Cwe>). Prion inoculations were performed by injecting different prion strains (RML6 and ME7) intracerebrally as described previously (Zhu *et al*, 2019), with a dose corresponding to 3 × 10^5^ LD_50_ on 8‐week‐old mice. Scrapie was diagnosed by the following set of clinical criteria: ataxia, kyphosis, priapism, and hind leg paresis. Mice were sacrificed on the day of the onset of the clinical signs of scrapie. As control non‐infectious brain homogenate (NBH) was intracerebrally injected into the mice, they were sacrificed approximately at the same time as when the prion‐infected mice reach the terminal stage (at the latest time point compatible with humane euthanasia) of the disease.

### Human samples

All human brain tissue samples used in the current study were anonymized and collected before 2010 and stored in a registered prion biobank at the University Hospital of Zurich. The study using the human brain tissue samples to monitor the levels of PIKfyve was approved by Kantonale Ethikkommission, Kanton Zürich (Permit ID: 2019‐02431).

Brain lysates from post‐mortal CJD and control brain tissues were generated by slicing a small region of frontal cortex followed by homogenization in lysis buffer (10 mM NaCl, 100 mM Tris, 10 mM EDTA, 0.5% sodium deoxycholate, 0.5% NP‐40, pH adjusted to 7) using a TissueLyser LT. Total amount of protein was estimated using bicinchoninic acid (BCA) assay, 30 µg of total protein was migrated on 4–12% SDS–PAGE, and Western blot was performed using anti‐PIKfyve antibody.

#### Reagents

A detailed list of all antibodies and reagents used in the current study along with the catalog numbers can be found in Table EV3.

### Cell culture and prion infection of cells

Gt1 cells were grown in Dulbecco's modified eagle medium (DMEM) in the presence of 10% fetal bovine serum (FBS), penicillin–streptomycin, and GlutaMAX (all obtained from Invitrogen). For prion infection of the cells, Gt1 cells growing in DMEM medium were incubated with either Rocky mountain laboratory strain of prion (RML6) prions (0.1%) or non‐infectious brain homogenate (NBH; 0.1%) for 3 days in one well of a 6‐well plate. This was followed by splitting the cells at 1:3 ratio every 3 days for at least 10 passages. The presence of infectivity in the cells was monitored by the presence of proteinase K (PK) resistant PrP, which can be detected by immunolabeling with POM1 antibody. At 70 dpi, the cells started developing vacuoles which were visualized by phase‐contrast microscopy. Transduction of lentivirus expressing GADD34 (1 × 10^8^ TU) was performed on chronically prion‐infected cells at 75 dpi. Cells were lysed on 79 dpi and processed for Western blotting.

### Quantification of vacuoles

Number of vacuolated cells were manually annotated and quantified using phase‐contrast microscopy. Four fields of vision were randomly chosen for each coverslip, and 250 cells were counted per field of vision. In total, 1,000 cells were counted per experimental condition and the total number of vacuolated cells is represented in the figures. A chi‐square test was carried out to estimate the statistical significance.

### Acyl‐rac assay

In the first step of the Acyl‐rac methodology, post‐nuclear supernatants were generated from the brains of terminally sick prion‐infected mice. Brains were homogenized in homogenization buffer (BH; 20 mM HEPES, pH 7.4, 200 mM NaCl, 1 mM dithiothreitol [DTT], 0.1 mM EDTA, and 0.3 mM PMSF) by using a Teflon glass homogenizer. The homogenate was subjected to centrifugation at 2,000 *g* for 15 min. The supernatant obtained was transferred into a separate Eppendorf. The amount of protein in the sample was estimated using a BCA assay, and 1 mg of the protein was subjected to ultracentrifugation at 200,000 *g* for 1 h. After centrifugation, the supernatant was discarded and pellet was resuspended in 100 µl of Buffer 1 (HEPES 25 mM, NaCl 25 mM, EDTA 1 mM, pH 7.4) in the presence of 0.5% Triton X‐100 and 1x protease inhibitors and vortex the sample. A further 200 µl of blocking buffer (HEPES 100 mM, EDTA 1mM, SDS 2.5%) was added to the sample along with 1.5% of MMTS and incubated at 40°C for 4 h with intermittent vortexing. 900 µl of ice‐cold acetone to the sample in the next step incubated at −20°C for 20 min. Centrifuge the samples at 5,000 *g* for 10 min and decant the supernatant. Wash the pellet with 70% acetone (five times) followed by centrifugation at 5,000 *g* for 10 min. Air dry the pellet and then re‐dissolve it in 400 µl of binding buffer (HEPES 100 mM, EDTA 1 mM, SDS 1.5%). Take 1/10^th^ of the sample (40 µl) as input. 150 µl of the sample is treated with freshly prepared hydroxylamine and 150 µl without any treatment. Incubate both tubes with 50 µl of the activated beads and leave the sample overnight on the rotating wheel at 4°C. Wash the beads with binding buffer (three times) followed by resuspension in the sample buffer (50 µl) with 10mM DTT. The samples were loaded on the gels, and Western blots were performed using anti‐PIKfyve antibody. For controls, antibodies against TRAPα and Calnexin were used.

To identify the DHHC enzyme responsible for acylating PIKfyve, Gt1 cells were transfected with siRNA against different DHHC (all from Qiagen) for 72 h followed by cell lysis to obtain post‐nuclear supernatant. Once the post‐nuclear supernatant was generated, the samples were treated in the exact way as described for the brain lysates.

### Western blot analysis

Mice brains were homogenized using TissueLyser LT for 5 min in 10 vol of lysis buffer (0.5% Nonidet P‐40, 0.5% 3‐[(3‐cholamidopropyl)dimethylammonio]‐1‐propanesulfonate (CHAPS), protease inhibitors (complete Mini, Roche), phosphates inhibitors (PhosphoSTOP, Roche) in PBS), and centrifuged at 1,000 *g* for 5 min at 4°C to remove debris. For Gt1 cells, lysates were prepared by exposing the cells to lysis buffer (PBS + Triton 1% + Protease inhibitors) for 15 min at 4°C followed by scraping off the cells from the plates. The lysates were centrifuged at 8,000 rpm for 10 min, and supernatant was used for further analysis. Protein lysates extracted from brains or cells were subjected to the standard BCA Assay (Thermo Fisher) to estimate the total amount of protein present, and in all cases, equal amounts of protein were loaded onto the SDS–PAGE (Novex NuPAGE 4–12% Bis‐Tris Gels). For PIKfyve, p‐TFEB, and TRPML1 Western blots, 80 µg of total brain/cell lysate was loaded on the gels. For all the other proteins, 40 µg of total protein was loaded onto the gels. Samples were transferred onto nitrocellulose membrane using iBlot (Invitrogen) according to the manufacturer's instructions. For proteins < 100 kDa, the transfer was performed at 20 V for 7 min. For proteins larger than 100 kDa, the transfer was performed at 15 V for 15 min. All samples were blocked in 5% SureBloc (lubio biosciences) for 1h followed by incubation with the primary antibody overnight in Tris‐buffered saline‐Tween (TBS‐T) at 4°C. Secondary Peroxidase‐Goat Anti‐Mouse IgG (H + L; #62‐6520) or Peroxidase‐Goat Anti‐Rabbit IgG (H + L; #111.035.045) used at 1:10,000 for 1 h at RT and membranes were developed using Luminata Crescendo (Millipore), and images were acquired using Fusion Solo 7S Edge (Vilber).

### Cerebellar organotypic cultured slice preparation

Cerebellar organotypic cultured slices (COCS) were generated from 9‐ to 12‐day‐old pups with a thickness of 350 µm as described previously (Falsig & Aguzzi, 2008). Cultures were maintained in a standard cell incubator (37°C, 5% CO_2_, 95% humidity), and the culture medium was changed three times per week. For prion inoculation of COCS, freshly generated slices were treated with 100 µg of prion‐infected brain homogenate for every 10 slices followed by culturing in a 6‐well Millicell‐CM Biopore PTFE membrane insert (Millipore) according to previously published protocol (Falsig *et al*, 2012). Infected slice cultures were maintained for a maximum of 50 days when neurodegeneration is prominent in the slices. For treatment with GSK2606414, slices were incubated with 20 µM of the drug for 14 days with a change in medium and replenishment with a fresh stock of drug every 3 days. On Day 35, the COCS were subjected to lysis and protein and RNA were isolated for further experiments.

For prion mimetics, toxicity in slices was induced by exposure to toxic anti‐PrP^C^ antibodies targeting the globular domain, such as single chain POM1 antibody, after a 14‐day recovery period, according to previously published protocol (Sonati *et al*, 2013). *tg*a*20* COCS were exposed to POM1 (67 nM), or as a control with IgG (67 nM) for 4 days and cell lysates for Western blots were generated in the lysis buffer (PBS + Triton 1% + protease inhibitor cocktail cOmplete mini, Roche).

### Immunofluorescence

Gt1 cells were grown on coverslips (10 mm) and on eight‐well culture slides (BD Biosciences) for prion‐infected cells, washed with ice‐cold 1× PBS, and fixed with 4% paraformaldehyde (PFA) in PBS, pH 7.4, for 15 min at RT. After washing 2× with ice‐cold PBS, cells were permeabilized with 0.1% Triton X‐100 in PBS for 10 min at RT followed by rinsing in 1× PBS for three times and incubated for 1 h in blocking solution (10% FBS in PBS) at room temperature. Cell was incubated in primary antibodies prepared in blocking buffer for 1 h followed by washing with blocking buffer (3×) and further incubation with Alexa‐conjugated secondary antibodies (Invitrogen, Molecular Probes), prepared in the blocking buffer at a dilution of 1:5,000 for 30 min along with 1 μg/ml DAPI (4’,6‐diamidino‐2‐phenylindole, Invitrogen). Samples were mounted onto the glass slides with fluorescent mounting medium (Dako). In the case of prion‐infected cells, glass slides were tightly sealed from the outside environment using nail polish. Samples were imaged using a CLSM Leica SP5 ZMB. Images were processed and analyzed using the “ImageJ” software.

### Transmission electron microscopy

Transmission electron microscopy was performed as previously described (Nuvolone *et al*, 2016). COCS samples were fixed *in situ* with 2.5% glutaraldehyde + 2% paraformaldehyde in 0.1 M phosphate buffer, pH 7.4, and embedded in Epon. Ultrathin sections were mounted on copper grids coated with Formvar membrane and contrasted with uranyl acetate/lead citrate. Micrographs were acquired using a Hitachi H‐7650 electron microscope (Hitachi High‐Tech, Japan) operating at 80 kV. Brightness and contrast were adjusted using Photoshop.

### Flow cytometry

Gt1 cells were plated in six‐well plates at a dilution of 250,000 cells/well and were transfected with either a control siRNA or siRNA targeting PIKfvye the following days. Cells were left in the medium containing siRNA for up to 72 h, and the generation of the vacuoles was visualized using phase‐contrast microscopy. Cells were washed 1× with FACS buffer (PBS + 10% FBS) followed by incubation with PBS‐EDTA (2 mM) for 15 min for detachment. Once the cells were detached, pooled quadruplicates were resuspended in FACS buffer and subjected to centrifugation at 900 rpm for 5 min. The pellets were resuspended in FACS buffer and the samples proceeded to flow cytometry. Before sample acquisition, each sample was treated with 5 µM LysoSensor Yellow/Blue (LysoSensor™ Yellow/Blue DND‐160, Thermo Fisher, #L7545) for 4.5 min at 37°C according to the manufacturer's guidelines. Consecutively, 5 µl of 1:5,000 pre‐diluted SYTOX Red dead cell stain (SYTOX™ Red dead cell stain, Thermo Fisher, #S34859) was added to each sample, shortly vortexed, and further incubated for 30 s followed by immediate sample acquisition and recording. Acquisition was performed using a BD FACSAria™ Fusion. Optical configurations were set as follows. A 355 nm UV and a 633 nm Red laser were used for optimal excitation of LysoSensor Yellow/Blue and SYTOX Red dead cell stain, respectively. The emission of LysoSensor Yellow/Blue was recorded using a LP502 mirror in combination with a BP530/30 filter and a LP410 mirror in combination with a BP450/20 filter. The emission of SYTOX Red was recorded using a BP670/30 filter. The flow cytometry data were analyzed using FlowJo 10.6.1. Gt1 cells were first gated for singlets and for living cells (SYTOX Red negatives) followed by further depiction in BP530/30 (acid) and BP450/20 (neutral/basic) histograms. The ratio of the mean fluorescence intensity (MFI) 530/30 of LysoSensor‐stained minus MFI 530/30 LysoSensor‐unstained and MFI 450/20 of LysoSensor‐stained minus MFI 450/20 LysoSensor‐unstained cells was calculated and plotted for the time points 24, 48, and 72 h post‐transfection (Fig 4E).

### Radiolabeling assays

To monitor the half‐life of PIKfyve, NBH or prion‐infected Gt1 cells (75 dpi) were incubated in the starvation medium (DMEM without methionine and cysteine) for 40 min to deplete the endogenous stores of methionine and cysteine. Cells were then labeled with 50 µCi/ml ^35^S‐methionine/cysteine for 20 min followed by a chase in normal medium for different time points. After the chase, the cells were harvested an isotonic HEPES buffer (pH 6.8) containing 2% CHAPS and protease inhibitor cocktail. Post‐nuclear supernatants were obtained by centrifuging the sample at 10,000 *g* for 10 min. PIKfyve was immunoprecipitated with anti‐PIKfyve antibody (Sigma) followed by incubation with Protein G Dynabeads (Invitrogen) for 2 h at 4°C. The immunoprecipitates were migrated on a 4–12% Tris‐BIS gels followed by fixation and drying of the gels. The dried gels were exposed to phosphoscreen, and the radiolabeled products were revealed using a Phosphorimager.

To monitor the translation rates of PIKfyve in prion infection and thapsigargin (500 nM for 4 h) treatment, Gt1 cells were incubated in the starvation medium for 40 min and labeled with 50 µCi/ml of ^35^S‐methionine/cysteine for 40 min, followed by cell lysis. PIKfyve was immunoprecipitated, and newly synthesized and radiolabeled PIKfyve was identified using a Phosphorimager.

To monitor palmitoylation, Gt1 cells expressing GFP‐PIKfyve or GFP‐PIKfyve Acyl were incubated with OptiMEM medium (buffered with 10 mM HEPES, pH 7.4) with 250 µCi/ml of ^3^H palmitic acid (Hartmann Analytic) for 3 h. Cells were lysed, and immunoprecipitation was performed using anti‐GFP antibody followed by incubation with Protein G Dynabeads. Immunoprecipitates were migrated on SDS–PAGE, dried gels were exposed to photoscreen, and radiolabeled products were identified using Phosphorimager.

### Immunoprecipitation

Protein extraction from prion‐infected or C57BL/6 mice brains was performed using mechanical lysis in IP buffer (HBS buffer [pH 6.8] with 2% CHAPS and cocktail of protease inhibitors [Roche]). Once lysed, the samples were subjected to centrifugation at 10,000 *g* for 10 min. Protein content in the sample was estimated using BCA assay (with BSA as a standard), and 1 mg of the protein was used for the immunoprecipitation assays. For the cell lines, lysis was performed for 20 min at 4°C in IP buffer (1% Triton X‐100 in PBS, Protease inhibitors 1×) followed by centrifugation for 10 min at 10,000 *g*. In both cases, supernatants were precleared and incubated for 16 h at 4°C with antibodies and Protein G Dynabeads (Invitrogen). After immunoprecipitation, the beads were washed for three times with the IP buffer and resuspended in the sample buffer (2×) after the final wash. The samples were heated at 95°C for 5 min and migrated on 4–12% Tris‐Bis gels with the MOPS buffer.

### Statistical methods and figure preparation

Error bars in all the figures represent standard error mean (SEM). All graphs were generated using GraphPad Prism.

### RNAi and shRNA

Gt1 cells were plated at a density of 500,000 cells/well of a six‐well plate and 24 h later were washed with PBS and replenished with medium without antibiotics. siRNA (final conc of 25 pmol/ well) was transfected into the cells using RNAi Max reagent (1%) according to manufacturer's instructions. 72 h later, cells processed to either obtain RNA or protein lysates or fixed for staining followed by imaging. List of siRNA used in the current study can be found in Table EV4.

shRNA targeting PIKfyve was generated by cloning the target sequence (5′‐GGCTTATGTATGCTTGATG‐3′) into pSuper.retro.puro plasmid between BglII and Xho1 sites. Gt1 cells were transfected with shRNA against PIKfyve followed by antibiotic selection (puromycin; 3 µg/ml for 24 h) of the transfected cells. At 144 h post‐transfection, cells were fixed and stained using anti‐TRPML1 antibody. For the vacuolation rescue experiment, cells were supplemented with bPIP (20 µg/ml) at 120 h post‐transfection with shRNA against PIKfyve for 24 h followed by manual quantification of number of vacuolated cells.

### Hexosaminidase beta assay

A fluorimetric assay was performed to detect the amount of Hexosaminidase beta in the samples using the manufacturer’s instructions in Beta Hexosaminidase Activity Assay kit from Cell Biolabs. The assay was based on the principle that in the presence of hexosaminidase beta, the substrate *p*‐nitrophenol‐*N*‐acetyl‐beta‐d‐glucosaminide is converted to *p*‐Nitrophenol which can be measured at 450 nm. 50 µg of brain lysates from control mice and prion‐infected mice in a final volume of 50 µl was incubated with 50 µl of substrate solution at 37°C for 15 min followed by addition of neutralization solution (100 µl). For cell lysates from prion‐infected and control Gt1 cells, 100 µg of cell lysate was used.

### mRNA isolation and quantitative real‐time PCR

Total RNA from brains of prion‐infected and controls was isolated using RNeasy Plus Universal Mini Kit (Qiagen), according to the manufacturer’s manual. After reverse transcription (QuantiTect Rev. Transcription Kit, Qiagen), cDNA was processed for real‐time qPCR using SYBR‐green (Roche) and determination of ΔΔCT‐values was done on a ViiA 7 real‐time system (Applied Biosystems). Total RNA from Gt1 cells infected with prions was isolated using RNeasy Mini Kit (Qiagen), according to the manufacturer's manual. RNA levels of GAPDH were used to standardize expression levels. RT–PCR was performed using SYBR‐green (Roche), and determination of ΔΔCT‐values was done on a ViiA 7 real‐time system (Applied Biosystems). For the primer sequences used in this study, see Table EV2.

### Secondary antibodies

All HRP‐tagged secondary antibodies were obtained from Jackson laboratories, and all Alexa‐tagged fluorescent secondary antibodies used for immunofluorescence and IHC were obtained from Invitrogen.

## Author contributions

AA and AKKL designed the experiments and wrote the manuscript. AA initiated and supervised the project. AKKL performed, or contributed to, all experiments including Western blots, immunoprecipitations, acylation assays, qPCR assays, experiments on cerebellar organotypic cultured slices (COCS), immunofluorescence, imaging, and vacuolation rescue experiments. EL performed imaging on prion‐infected cells, ER stress experiment in GT1 cells, and alternative splicing of PIKfyve. KF contributed to the Western blots on human CJD samples, performed the bodipy‐PI(3,5)P_2_ rescue experiments on RML treated and POM1‐treated slices, performed quantifications on slice cultures, and wrote the bioethical application to perform the experiments on human samples. UH contributed to the preparation of COCS, prepared the COCS for the rescue of PIKfyve levels upon treatment with GSK2606414, treated the mice with Lin5044 LCP and generated the brain lysates, and generated shRNA against PIKfyve. ML performed the FACS experiment to monitor the content of the vacuoles. RM set up conditions for performing Western blots using various antibodies generated the prion‐infected Gt1 cells, performed Western blots, IP, staining of infected Gt1 cells, contributed to the bodipy‐PI(3,5)P_2_ rescue experiment in Gt1 cells, and performed immunofluorescence on prion‐infected cells and their decontamination for imaging. All authors approved the final version of the manuscript.

## Conflict of interest

The authors declare that they have no conflict of interest.

## Supporting information



Appendix

Table EV1

Table EV2

Table EV3

Table EV4

Source Data for Appendix

Source Data for Figure 1

Source Data for Figure 2

Source Data for Figure 3

Source Data for Figure 5

Source Data for Figure 6

## Data Availability

Uncropped Western blots from the entire manuscript are included in Source data files. Any additional information/data required will be made available by the corresponding author upon reasonable request. This study includes no data deposited in external repositories.
