# Supplementary material for: Loss of PIKfyve drives the spongiform degeneration in prion diseases
Source: EMBO Mol Med. 2021 Jul 22;13(9):e14714. doi: 10.15252/emmm.202114714 (PMC8518562; doi:10.15252/emmm.202114714)
Supplement: Supplementary file 1 — Appendix [file EMMM-13-e14714-s012.pdf]

Appendix Figures

Lakkaraju et al; Loss of PIKfyve drives the spongiform degeneration in prion diseases.

Table of Contents

Appendix Figure S1---2

Appendix Figure S2---4

Appendix Figure S3---6

Appendix Figure S4---8

Appendix Figure S5---9

Appendix Figure S6---11

Appendix Figure S7---12

Appendix Figure S1

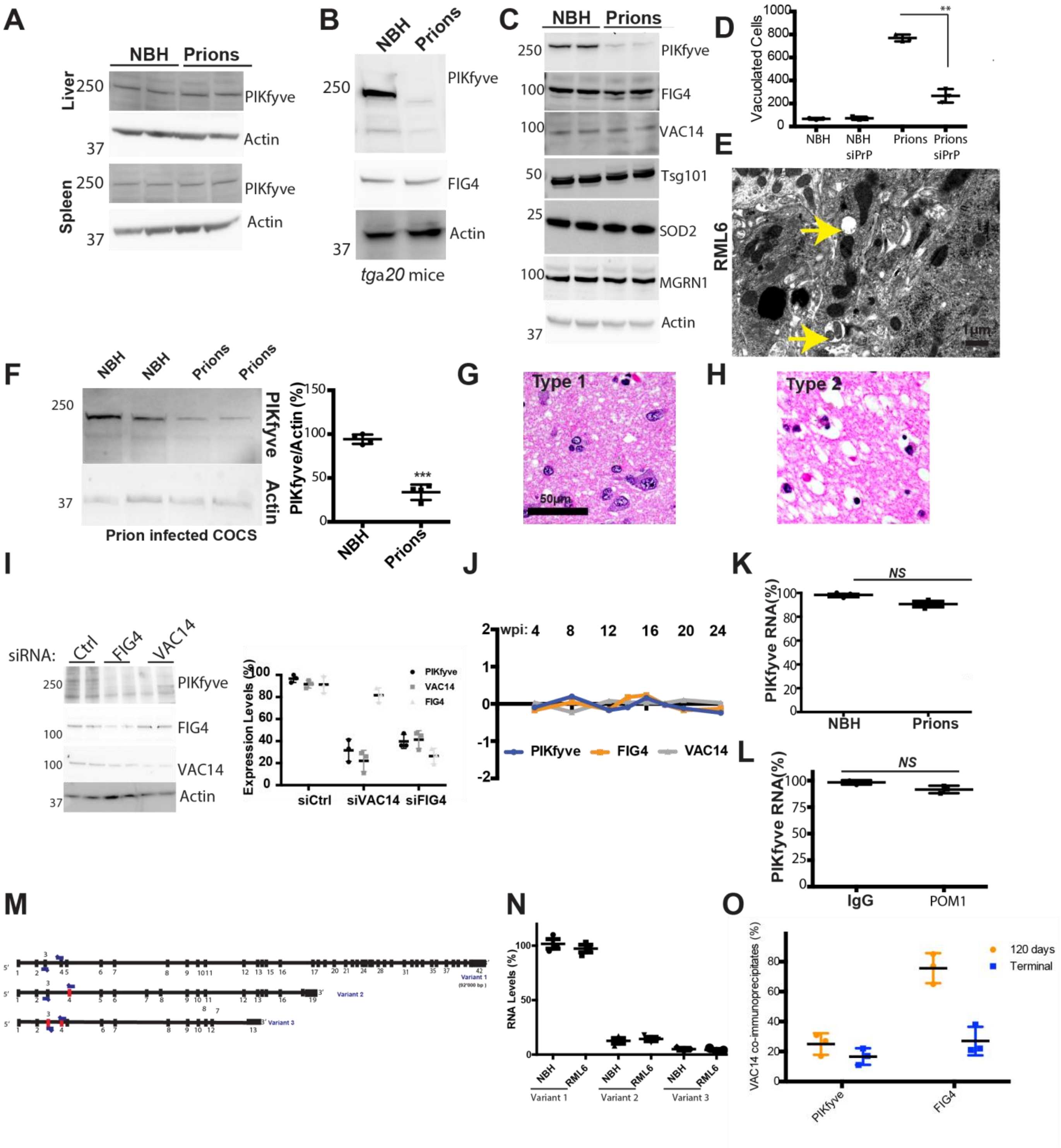

A: Liver and spleen lysates were prepared from terminally sick C57BL/6 mice infected with RML6 prions and, for control, age-matched mice injected with NBH. Samples (50 µg) were subjected to SDS-PAGE followed by Western blot with antibodies against PIKfyve and actin. In both organs PIKfyve levels were unaffected by prion infection. N=3. **B:** Brain lysates were prepared from terminally scrapie-sick *tga20* mice (overexpressing PrP^C) infected with RML6 prions. For control we used age-matched mice inoculated with NBH. Western blot analysis revealed downregulation of PIKfyve but no effect on FIG4. Actin: loading control. N=3. **C:** Brain lysates were prepared from terminally sick C57BL/6 mice infected with RML6 prion. For control we used age-matched mice

inoculated with NBH. Samples (50 µg) were subjected to SDS-PAGE followed by Western blot with antibodies against PIKfyve, FIG4, VAC14, Tsg101, SOD2, MGRN1 and actin. N=3. Only PIKfyve was significantly downregulated in prion infection ($p<0.001$); Statistics: ANOVA. **D:** Gt1 cells infected with RML6 prions or NBH were treated with siRNA against PrP^{C} for 96h at 70dpi. Phase contrast microscopy revealed a significant decrease in the number of vacuolated cells upon depletion of PrP^{C} in RML6 infected cells. 1000 cells were manually quantified for the presence of vacuoles. N=3. Statistics: Chi-Square test. **E:** Electron microscopy image showing membrane-lined microvacuoles (arrows) in prion-infected COCS at 56 dpi. The vacuoles contained degenerating organelles and other cellular debris. Yellow arrows: vacuoles. N=3. **F:** Cerebellar organotypic cultured slices (COCS) were generated from *tga20* mice pups and inoculated with RML6 or, as a control, with NBH. At 45 dpi, when neurodegeneration was prominent, COCS were lysed and equal amounts of the protein was used for western blot analysis. PIKfvye was depleted in RML6 infected COCS. Loading control: actin. Right: Quantification of the western blot shows a significant reduction in PIKfyve levels. Experiments were repeated three times independently. **G-H:** Representative hematoxylin and eosin (H&E) staining of the human cortical brain tissue obtained at autopsy from patients who suffered from Type-1 or Type 2-CJD. AMmore extensive vacuolation can be observed in Type-2 CJD. **I:** Gt1 cells were transfected with siRNA against VAC14 or FIG4. For control we used an siRNA containing a scrambled sequence. Cell were harvested 72 hours post transfection and 50 µg of protein was migrated on SDS-PAGE followed by Western blotting using anti PIKfyve, FIG4 and VAC14 antibodies. PIKfyve levels were reduced in cells with VAC14 and FIG4 downregulation. Quantification of western blots: Each dot represents an individual experiment. Statistics: Unpaired t test. **J:** The $\log_2$ fold changes in the mRNA expression of PIKfyve, FIG4 and VAC14 during the course of prion disease were extracted from a longitudinal database of transcriptional changes in prion disease progression [1]. None of the genes show significant alterations during the progression of the prion disease. **K:** cDNA generated from the lysates described in Fig. S1E was subjected to qPCR using primers targeting PIKfyve. No change was observed in the expression levels of PIKfyve after prion infection. N=3. **L:** Total RNA was isolated from COCS treated for 72 hours with POM1 or IgG for control. As a further control we used POM1 preincubated with recombinant PrP (thereby blocking its paratope). RNA was retrotranscribed, and cDNA was subjected to qPCR using primers against PIKfyve. PIKfyve RNA levels remained unaltered after treatment with POM1 N=3. **M:** Genomic map of the three differentially spliced isoforms of PIKfyve. Vertical lines denote the exons (not to scale). Variant 1 represents the protein coding and the major isoform and consists of 42 exons. Variant 2 and 3 are the splice variants which contain exons which result from differential splicing (marked in red). Specific primers (marked in blue) were used spanning the exons that are specific to each of the isoform measure their levels in brains using real time quantitative PCR (qPCR). **N:** Total RNA was isolated from the brains of terminally sick C57BL/6 mice infected with RML6 prions and as a control RNA was isolated from NBH infected mice (same samples as in D). 1 µg of RNA was subjected to reverse transcription to generate cDNA followed by real time quantitative PCR (-PCR) using primers targeting each of the three isoforms of murine PIKfyve individually. Variant 1 represents the full length and is predominant isoform expressed in the brains. Variant 2 and 3 are expressed in low amounts in comparison to variant 1. The levels of all the isoforms were unaffected by prion infection. Each dot represents an individual mouse; Statistics: ANOVA. O: Quantification of western blot from Fig. 1J. PIkfyve complex is disrupted at the terminal stage of the disease. Each dot represents an individual biological replicate. Statistics: unpaired t-test

Appendix Figure S2:

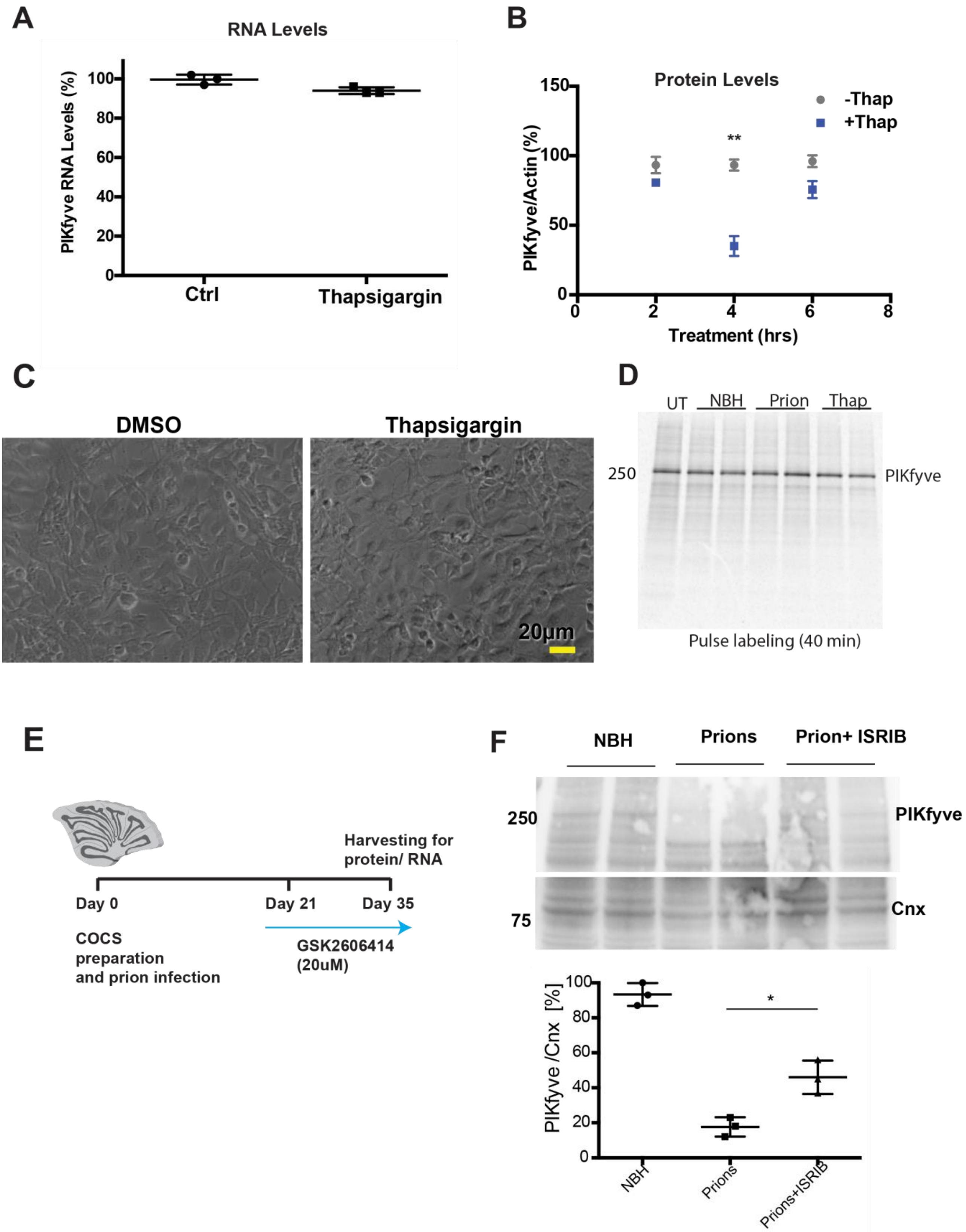

A: RNA was isolated from Gt1 cells treated with thapsigargin (0.5 µM for 4h), retrotranscribed and subjected to quantitative real-time PCR. PIKfyve mRNA remained unaltered. Panels depict three independent experiments. Statistics: Unpaired t-test. **B:** Quantification of western blot from Fig. 2D. The experiment was repeated three independent times. PIKfyve levels significantly decrease 4 hours post thapsigargin treatment but recover back at 6 hours post treatment. **C:** Gt1 cells were treated with thapsigargin (0.5µM for 4hours) followed by imaging using a phase contrast microscope at 20x

magnification. N=3. **D:** Gt1 cells were inoculated with NBH / prions (75 dpi) or treated with thapsigargin (500nM, 4 hours), and metabolically pulsed with [^{35}S]Met/Cys for 40 min at 37°C. Cells were lysed and proteins were immunoprecipitated using anti-PIKfyve antibody. Immunoprecipitates were subjected to SDS-PAGE and autoradiography. There was no discernible change in the translation rate of PIkfyve upon prion infection or induction of UPR. Experiments were repeated three times independently. **E:** Schematics of GSK2606414 treatment of prion infected COCS. Prion-infected COCS were continuously treated with GSK2606414 starting from day 21 until day 45 when the samples were lysed, and protein and RNA was harvested for further analysis**. F:** Prion infected Gt1 cells (70dpi) were treated with ISRIB (300mM) for 5 days and samples were lysed and western blot was performed using anti-PIKfyve antibody. As control NBH treated cells were used. Quantification: Each dot represents an individual experiment. Statistics: Unpaired t-test.

Appendix Figure S3

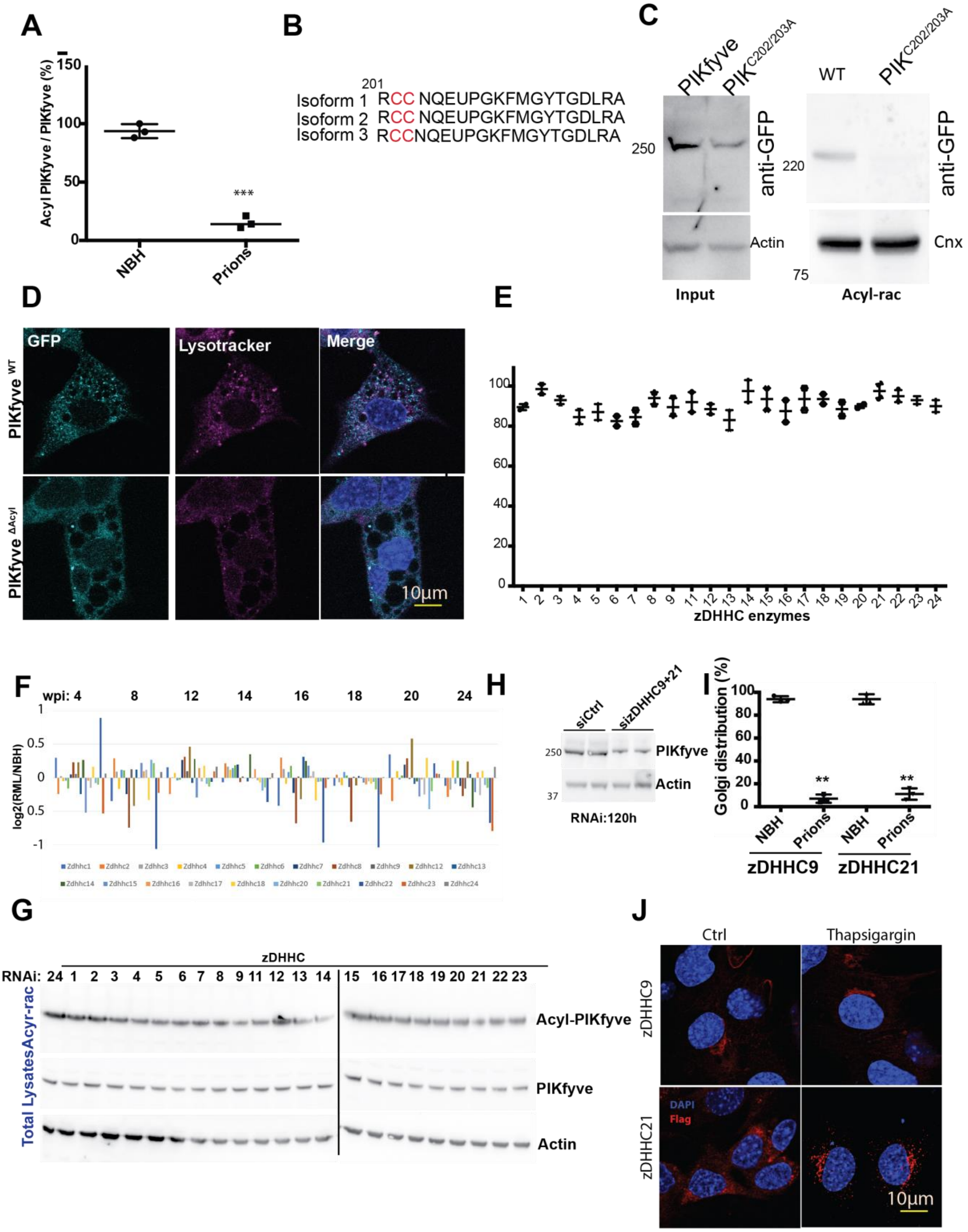

A: Quantification of western blot from Fig. 3B. Acylated PIKfyve levels were significantly downregulated in prion infected mice at 60 dpi. Each dot represents an individual mouse. Statistics: Unpaired-t test. **B:** Sequence alignment of all the three isoforms of murine PIKfyve revealed the presence of conserved cysteines at positions 203 and 203, which were predicted by the acylation predictor to be the potential palmitoylation sites in PIKfyve. **C:** Gt1 cells were transiently transfected with full length wild type PIKfyve cDNA (PIKfyve^{WT}) or with PIKfyve plasmid where the cysteines at position 202/203 were replaced with alanines (PIKfyve$^{\Delta Acyl}$). 48h post transfection, cell lysates were subjected to acyl-rac and immunoblotted with anti-GFP antibody. PIKfyve$^{\Delta Acyl}$ was not acylated (right panel) and the total lysates revealed a lower expression of PIKfyve$^{\Delta Acyl}$ (left panel). Calnexin was used an acylation control and as loading control. Experiments were repeated three times independently.**D:** Gt1 cells were transiently transfected with PIKfyve-GFP or PIKfyve$^{\Delta Acyl}$ for 24h followed by treated with lysotracker for 60 min. Immunostaining revealed generation of extensive vacuoles in both conditions. Experiments were repeated three times independently. **E:** Total RNA was isolated from the brains of terminally sick C57BL/6 mice infected with RML6 and subjected to reverse transcription. As control RNA from NBH infected mice was used. cDNA was subjected to qPCR using specific primers targeting each DHHC enzyme. No change in the levels of mRNA encoding any of the DHHC enzymes was observed between RML6-infected and NBH-exposed samples. Each dot represents an individual experiment. **F:** The $\log_2$ fold changes in the mRNA expression of all zDHHC enzymes during the course of prion disease were plotted from the data available on transcriptional changes in prion disease progression[2]. None of the genes show significant alterations during the progression of the prion disease. Multiple mice were used for the analysis. **G:** Gt1 cells were transfected with siRNA against several zDHHC enzymes. After 72 hours, lysates were subjected to Acyl-rac and immunoblotted for PIKfyve. siRNA against zDHHC9 and 21 resulted in modest decrease in PIKfyve acylation. Lower panel represents the input (1/10th of the sample used from Acyl-rac). The experiment was repeated two times independently to confirm the identity of zDHHC responsible for acylating PIKfyve. **H:** Gt1 cells were treated with either a siRNA cocktail targeting zDHHC9/21 or a control siRNA (non targeting siRNA). 120 hours post transfection, cells were lysed and immunoblotted for PIKfyve. PIKfyve levels decreased in the prolonged absence of zDHHC9/21. **I:** Quantification of Golgi localization of zDHHC9 and 21 in prion infected cells from Fig.3H-I. A significant reduction of golgi localization of zDHHC9/21 in prion infected vacuolating cells was observed. Each dot represents an independent experiment (25 images were quantified per experiment). Statistics: unpaired t-test. **J:** Gt1 cells were transiently transfected with either flag tagged zDHHC9 or zDHHC21 plasmids for 24h followed by treatment with thapsigargin for 4 hours. Cells were immunostained using anti flag antibody. Images did not reveal mislocalization of zDHHC9 or zDHHC21 upon induction of UPR.

Appendix Figure S4.

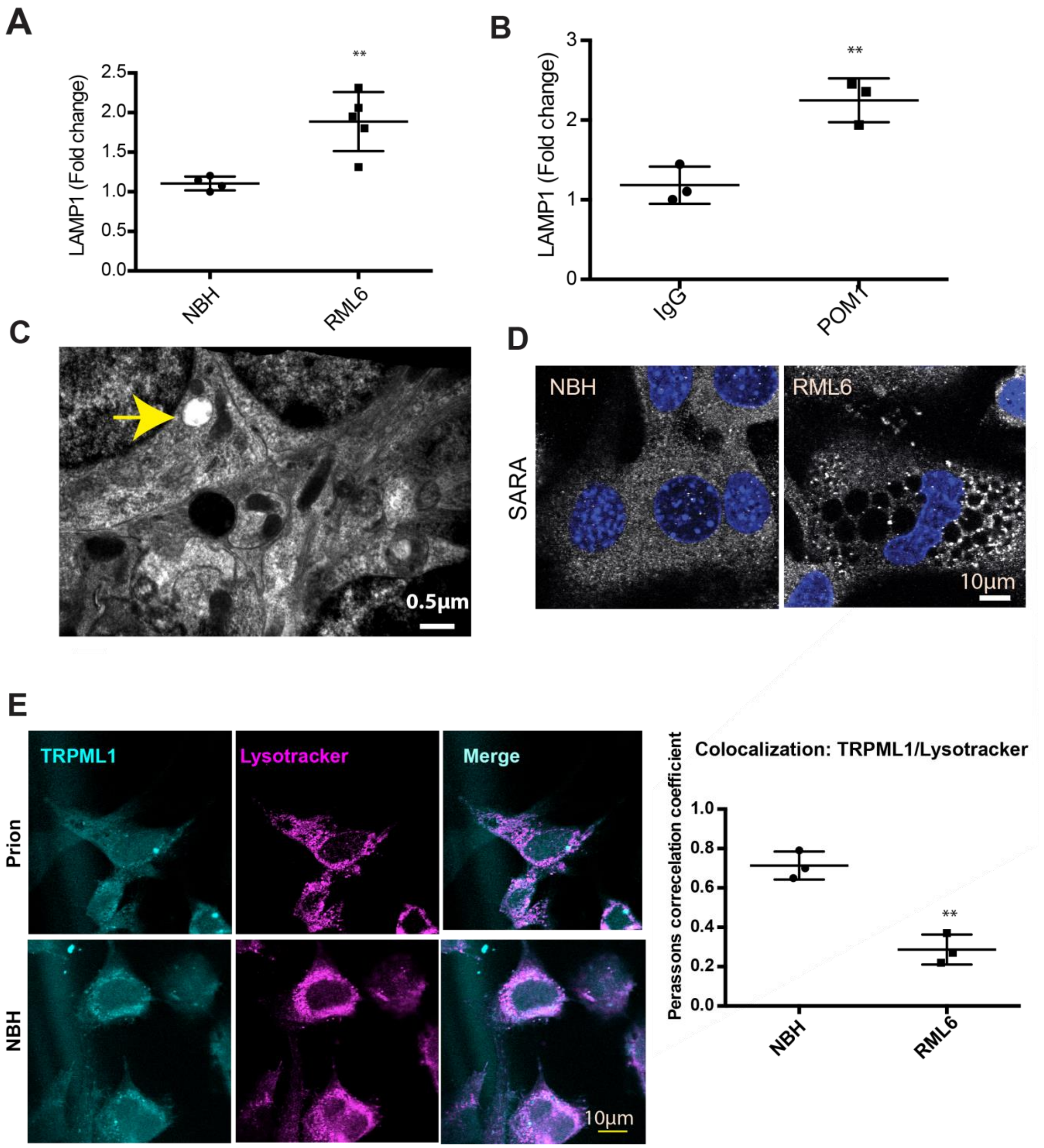

A-B: Quantification of LAMP1 fluorescence in RML6 infected and POM1 treated *tga20* COCS. Only NeuN positive cells were considered for quantification. N=9. Statistics: unpaired t test. **C:** Electron microscopy image of COCS treated with POM1 for 15 days. Microvacuolation without any discenible intracellular material was observed (N=3). **D:** Prion infected Gt1 cells (75dpi) were fixed and immunostained with anti-SARA antibodies. As controls NBH treated cells were used. SARA lines the vacuoles similar to LAMP1 (N=3). **E:** Gt1 cells were infected with prions or treated with NBH as a control. At 75 dpi, cells were treated with lysotracker for 20 min followed by fixation and immunostaining with TRPML1 antibody. Colocalization analysis revealed decreased TRPML associated with lysotracker. Quantification: Pearson correlation coefficient of colocalization was calculated for TRPML1 and lysotracker. The analysis revealed a decreased correlation in prion infected cells. Each dot represents an individual biological replicate, and the 25 images were used for quantification per replicate. Statistics: Unpaired t-test.

Appendix Figure S5

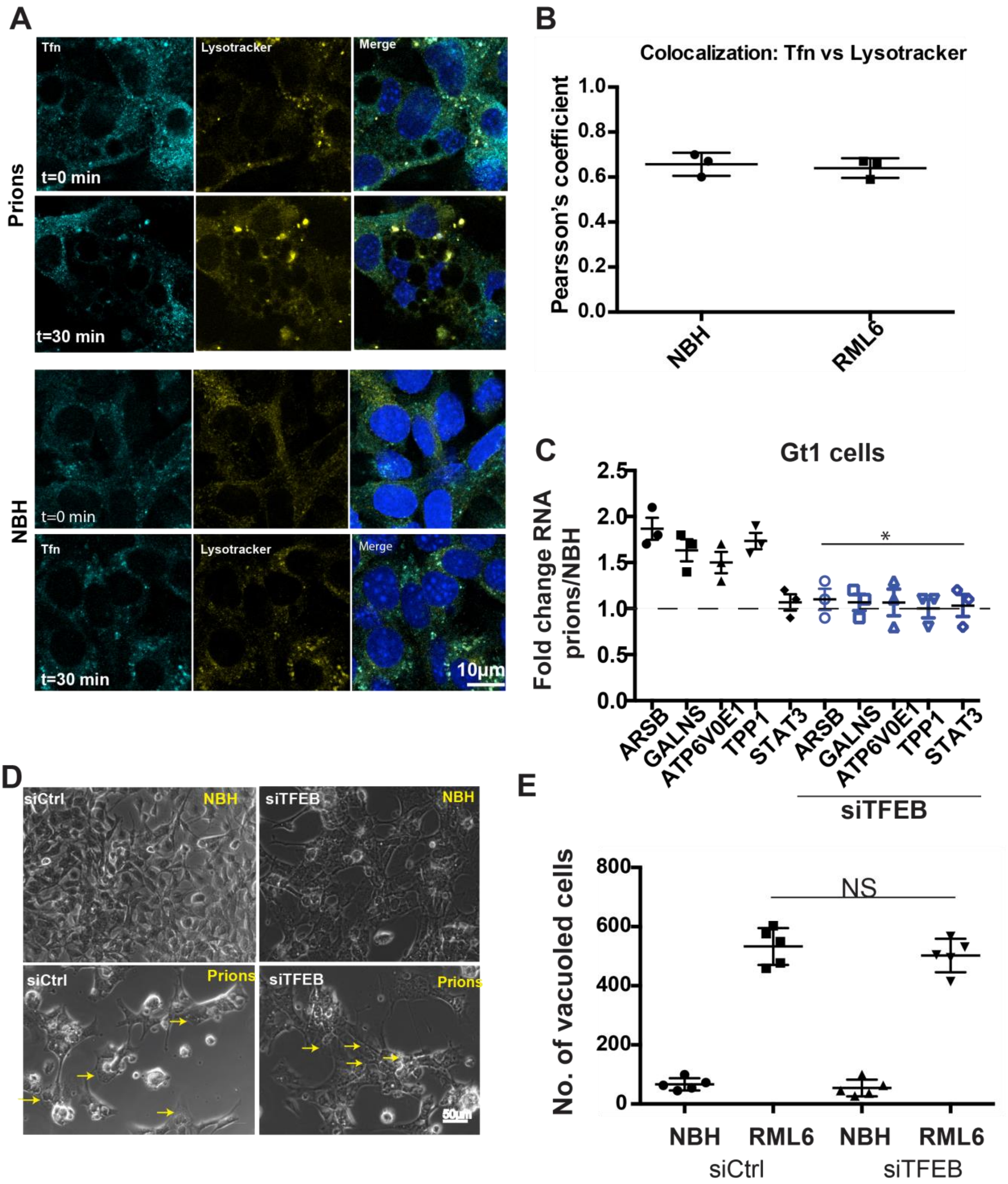

A: Gt1 cells were infected with prions or treated with NBH (75dpi). Cells were pulsed with Alexa 488-transferrin (10µg/ml**)** for 20 min and were either fixed immediately or chased in CO2 free medium for 30 min. Lysotracker was added into the pulsing medium to detect acidic lysosomes. Alexa 488 transferrin and lysotracker staining were pseudo colored to cyan and yellow respectively (DAPI: blue)

B: Pearsson correlation coefficient was used to calculate the colocalization between Alexa-488 transferrin and lysotracker staining. Each dot represents an individual biological replicate. Quantification (25 images per replicate) revealed colocalization of Alexa488 transferrin with lysosotracker in prion infected cells suggesting that endocytosis is not altered in prion infection
C: Gt1 cells with infected with RML6 and 70dpi, cells were treated with siRNA against TFEB for 5 days and were analyzed for lysosomal gene homeostatic levels using qPCR. TFEB downregulation resulted in restoration of lysosomal genes to their homeostatic levels. As control NBH treated cells were used. ARSB: Arylsulfatase B, GALNS: Galactosamine-6-sulfatase, ATP6V0E1: ATPase H^+-transporting-V0-Subunit-E1, TPP1: Tripeptidyl peptidase. Panels depict independent triplicates (Statistics: ANOVA). **D**: Phase contrast microscopy of cells in E. Vacuolation of prion-infected cells was not rescued by TFEB downregulation. Vacuoles: yellow arrows. **E:** Quantification of cells exhibiting vacuoles confirmed no rescue by TFEB downregulation. Each dot represents a separate experiment in which 1000 cells were counted. Statistics: Chi square test.

Appendix Figure S6

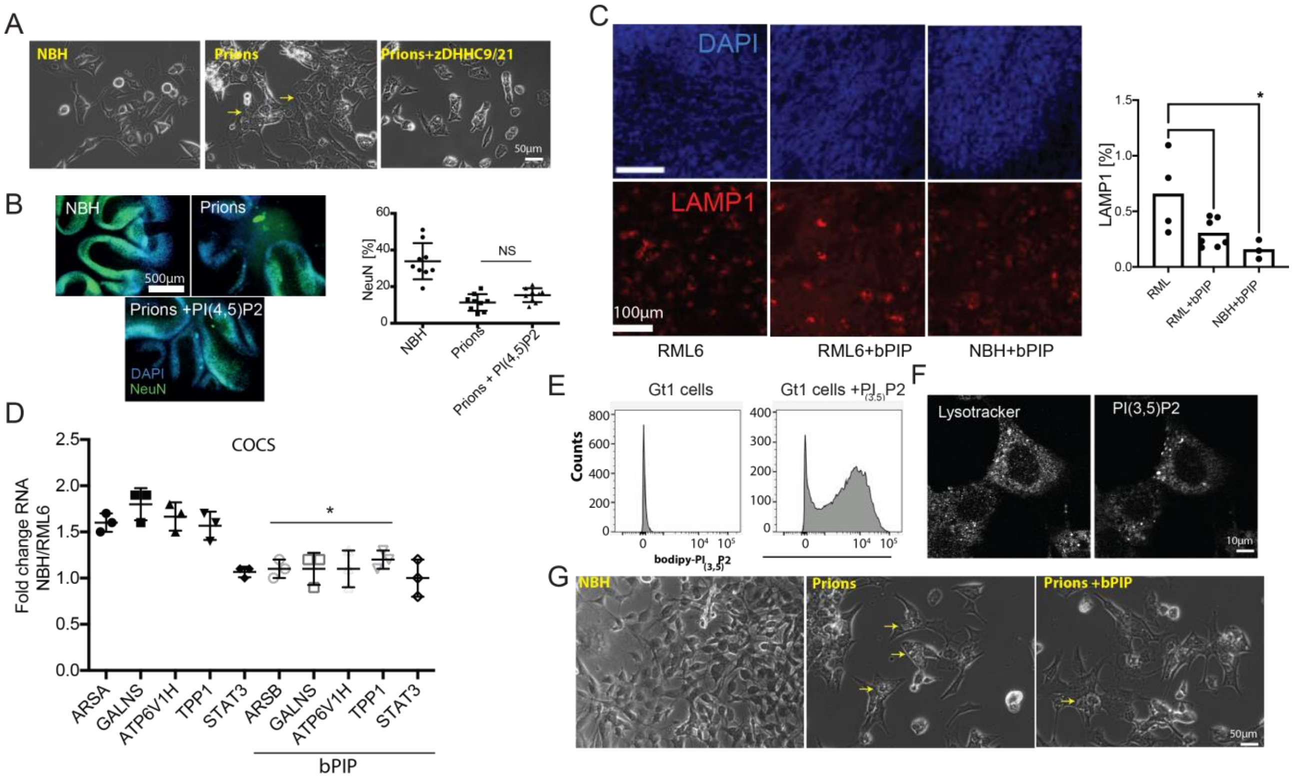

A: Representative phase contrast images from Fig. 6B. Overexpression if zDHHC9 and 21 in prion infected Gt1 cells partially rescues the levels of PIKfyve and vacuolation**.** **B:** *tga20* COCS were infected with prions and optionally treated with bodipy-PI(4,5)P2 (5 µg/ml). At 45 dpi, NeuN morphometry revealed ablation of cerebellar granule layer (CGN) in prion infected slices and PI(4,5)P2 treatment does not provide any rescue of NeuN loss. Control: NBH treated COCS. Each dot represents an individual slice (Statistics: ANOVA) **C:** *tga20* COCS were infected with RML6 and optionally treated with bPIP (5µg/ml, 45 dpi) followed by immunostaining with LAMP1. NBH-exposed bPIP-treated COCS were used for control. Prion-infected COCS showed increased $LAMP1^+$ pixels; the increase was depressed by bPIP. Each dot represents an individual COCS. Statistics: ANOVA. **D:** COCS from A were analyzed for lysosomal gene expression by qPCR. bPIP treatment prevented the upregulation of TFEB-responsive genes in prion-infected COCS. ARSB: Arylsulfatase B, GALNS: Galactosamine-6-sulfatase, ATP6V0E1: ATPase H^+-transporting-V0-Subunit-E1, TPP1: Tripeptidyl peptidase. Panels depict independent triplicates (ANOVA). **E:** Gt1 cells were incubated with bPIP (20µg/ml; 12 hours) and subjected to cytofluorimetric analysis. 62% of cells took up bPIP. **F:** Gt1 cells were treated with bPIP as in A and incubated with Lysotracker (1 hour). bPIP localized to lysosomal regions stained by Lysotracker. **G:** Gt1 cells were infected with RML6 and treated with bPIP (20µg/ml) for 5 days at 70 dpi. Depletion of PIKfyve resulted in vacuolation (left panel) which was significantly rescued by bPIP. Quantification shown in Fig. 6D; statistics: Chi-Square.

Appendix Figure S7

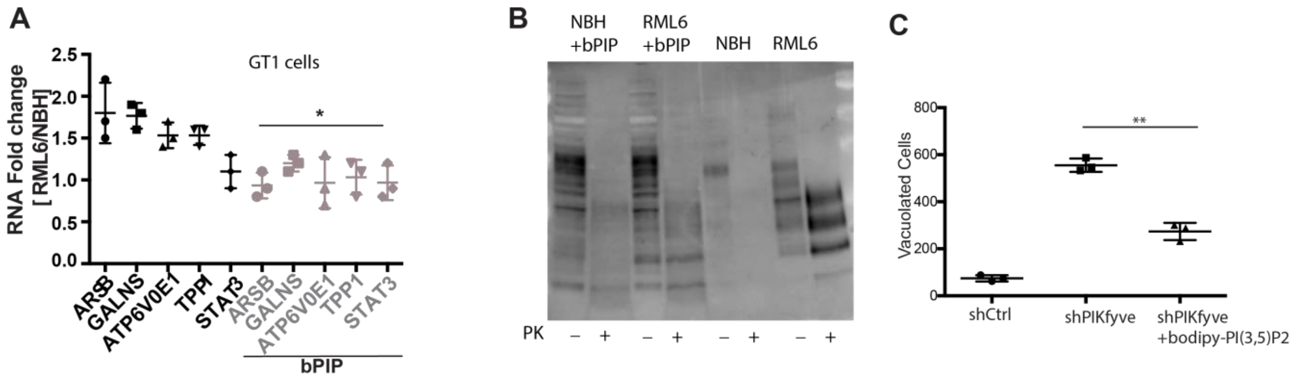

A: The Gt1 cell cultures depicted in Fig. 6G were analyzed for lysosomal gene expression by qPCR. bPIP treatment prevented the upregulation of TFEB responsive genes in RML6 prion infected Gt1 cells. ARSB: Arylsulfatase B, GALNS: Galactosamine-6-sulfatase, ATP6V0E1: ATPase H^+-transporting-V0-Subunit-E1, TPP1: Tripeptidyl peptidase. Panels depict independent triplicates (ANOVA). **B**: Representative Western blot from the prion infected Gt1 cells (Fig.6G) for total PrP^C and proteinase K resistant PrP^{Sc}. The presence of PrP^{Sc} in bPIP-treated cells suggests that bPIP acts downstream of prion aggregation. NBH-treated cells were used as controls. **C:** Gt1 cells were treated with shRNA against PIKfyve for 4 days in the presence or absence of bPIP (10 µM). 1000 cells were counted per experimental condition manually using phase contrast microscopy; each dot represents an individual experiment. Statistics: Chi-Square test.
