# Supplementary material for: Loss of PIKfyve drives the spongiform degeneration in prion diseases
Source: EMBO Mol Med. 2021 Jul 22;13(9):e14714. doi: 10.15252/emmm.202114714 (PMC8518562; doi:10.15252/emmm.202114714)
Supplement: Supplementary file 2 — Table EV1 [file EMMM-13-e14714-s010.docx]

**Table EV1:** Clinical and demographic information of human samples. Neither age (Pearson r = -0.08, p = 0.75) nor gender (mean female: 39.66 ± SD 21.87, mean male 48.2 ± SD 19.3, p = 0.41, unpaired two-sided T-test) of the patients correlated with cortical PIKfyve levels.

| Age | Gender | Clinical duration [months] | Diagnosis at autopsy | PrPSc type * | Co-morbidities |
| --- | --- | --- | --- | --- | --- |
| 67 | F | 6 | sCJD | 2 | none |
| 73 | F | n/a | sCJD | 2 | n/a |
| 69 | F | 1.5 | sCJD | 2 | Hashimoto's thyreoditis,  recurrent peripheral vein thrombosis,  disc protrusion S1 |
| 66 | F | n/a | sCJD | 2 | n/a |
| 58 | M | 2 | sCJD | 2 | n/a |
| 57 | M | 6 | sCJD | 1 | n/a |
| 57 | M | 13 | sCJD | 1 | paroxysmal atrial tachycardia |
| 72 | M | 2 | sCJD | 1 | Primary age-related tauopathy  (Braak&Braak Stage II) |
| 75 | M | 2 | sCJD | 1 | n/a |
| 74 | M | 2 | sCJD | 1 | ischemic insular stroke 4 months before death, coronary heart disease |
| 73 | M | n/a | sCJD | 2 | epidermoid carcinoma of the anal canal |
| 51 | F | 3 | sCJD | 2 | n/a |
| 64 | F | n/a | sCJD | 1 | n/a |
| 66 | M | n/a | sCJD | 1 | diabetes mellitus |
| 63 | M | 5 | sCJD | 2 | vitamin B12 deficiency, left frontal meningeoma |
| 54 | F | n/a | sCJD | 1 | n/a |
| 39 | M | 14 | sCJD | 1 | n/a |

| Gender | Age at Death | Cause of death |
| --- | --- | --- |
| M | 51 | Respiratory failure |
| F | 53 | Respiratory failure |
| F | 33 | Cardiac failure |
| F | 52 | Hypovolemic shock |

**Controls**

*According to **Parchi et al., Ann Neurol 1999**

n/a = no information available
