# Supplementary material for: Loss of PIKfyve drives the spongiform degeneration in prion diseases
Source: EMBO Mol Med. 2021 Jul 22;13(9):e14714. doi: 10.15252/emmm.202114714 (PMC8518562; doi:10.15252/emmm.202114714)
Supplement: Supplementary file 3 — Table EV2 [file EMMM-13-e14714-s008.docx]

**Table EV2**: List of primers used in the current study.

| **Gene name** | **Forward primer (5´-3´)** | **Reverse primer (5´-3´)** |
| --- | --- | --- |
| PIKfyve | AGTCTGTGAGGTCGCCTGTA | TCTGGAGTCTAACACTGAAGAGC |
| Zdhhc1 | ATCCTTCTGGGCCTGCTTTC | CATCTCCTGAATGGACCGCA |
| Zdhhc2 | TGCAGAGAAAGAATTGCTGGAG | CACAATATCGGATTGCGCCG |
| Zdhhc3 | CCTCCAGATTGACGATGCCA | CACACTTCTCTGGCTGGAGG |
| Zdhhc4 | CGGAAGTCTTGGGGAGCG | TCAGATAGCAGCTCCGCTTG |
| Zdhhc5 | GCACCATATCCCATCTGCATC | TCCCTGTTTCCAGGTTAGTCA |
| Zdhhc6 | CGACCTAGTCGACCCAGCATC | GAAACACAGCAGGACAAACGTC |
| Zdhhc7 | AAGCAACTTGTAAGGGTGTTTC | CCACGTCATGACAGCACAGA |
| Zdhhc8 | GCACACGCTGGTTAAGAAGG | GCAGATGAGTGGTGGTCAGT |
| Zdhhc9 | GACGTGAGGAGCGTTCCATT | GTGGTAGCGACTTCTCCCTG |
| Zdhhc11 | GTGGCATCAACAAGAACTGGG | AGGGCGTGGTAGGAACTTCT |
| Zdhhc12 | CTGCACGACACCGAGCTAC | GGGGTCCATGAGTGACACAG |
| Zdhhc13 | GATCAGTTGGGTGGTGACCT | CTGAAACAGGACCGCCAGAT |
| Zdhhc14 | GCCTCCTGTGTGACAATGAC | GGCTGTTTTGAATTATTCCTTACCA |
| Zdhhc15 | GATAGTCGAGATTATCCAGAAGGCT | TGGAACACCCGAACTGTGAC |
| Zdhhc16 | GACTGCTCACTCAGCCTCTG | CTGCCCTTGGAAGCTCTTGA |
| Zdhhc17 | CTGAAACGCTTTCTCCCAGC | CCCGTTTCGGTCTCGTACTC |
| Zdhhc18 | TGCAGTCTGATACCGCGTTG | CAGGTGGCAAGCACATTCAG |
| Zdhhc19 | GCGTGTTTGCTGCCTTCAAT | CCAGCCACCTACAAGGGAAT |
| Zdhhc20 | TGGACGAATGAACCAACAGT | CAACCAGCCAGCAATGGTAG |
| Zdhhc21 | GGCATGCCGACACCCACT | TCGTATGGGCCTCCCTTCAAT |
| Zdhhc22 | CCTCTACACCTCTCTGGCCT | CTCCGGAGAAGAACTGGCTG |
| Zdhhc23 | CAACAACCGCACACTGAAGG | ACAATGATGGTCCATTCTCCGT |
| Zdhhc24 | GCTTATCAGCTGCTCAATCTGC | GGTCACGACGTAGGATGCAC |
| Cathepsin A | TCAGGCAGTGAAAACTCGGG | CGGTTCCGGGCATGTCTTG |
| Cathepsin B | GCTCTTGTTGGGCATTTGGG | ACTCGGCCATTGGTGTGAAT |
| Glucocerebrosidase A | TGGAGAGAAGTGTGCTGGTG | CAGACCACTGAGCTGTAGCC |
| LAMP1 | GCCCTGGAATTGCAGTTTGG | TGCTGAATGTGGGCACTAGG |
| Galactosidase Alpha | CCCGAGAGGGATTCAAAGGG | TGTGGACGTAATTTGCGAGGT |
| Mucolipin 1 | TGCTGTGGACCAGTACCTGA | GTAGTACCGCTGGCAGAGAG |
| GALNS | CATGGACGATATGGGGTGGG | CTGCAGCCATCCGGTCTAAA |
| Aryl Sulfatase B | TGCGCCGATTGAGTCTTTGA | AACAGTGGTTTCTCCGGTGG |
| ATP6V0E1 | GGGTCCTAACCGGGGAGTTA | ACAGAGGATTGAGCTGTGCC |
| TPP1 | CTACTGGGTGGTCAGCAACA | CAGCCGTGGGTTACATCAAAG |
| STAT3 | GCAATACCATTGACCTGCCG | ACGTGAGCGACTCAAACTGC |
| βactin | CTGAGCTGCGTTTTACACCC | CGCCTTCACCGTTCCAGTTT |
| GAPDH | CCACCCCAGCAAGGAGAC | GAAATTGTGAGGGAGATGCT |
| PIKfvye variant1 | GCCACATCCTCAGGAGAG | GCGTTTCAATACTGTGCTG |
| PIkfyve variant-2 | CTCCAGAAGGAAAGCAG | CAGTAGGTGCATGTCGG |
| PIKfyve variant-3 | CATCCTCAGGAGAGCACAG | GAGGCGTTTCAATACTGTG |
