# Supplementary material for: Loss of PIKfyve drives the spongiform degeneration in prion diseases
Source: EMBO Mol Med. 2021 Jul 22;13(9):e14714. doi: 10.15252/emmm.202114714 (PMC8518562; doi:10.15252/emmm.202114714)
Supplement: Supplementary file 4 — Table EV3 [file EMMM-13-e14714-s005.docx]

**Table EV3**: List of all antibodies and reagents used in the current study.

| **Reagent or resource** | **Source** | **Identifier** |
| --- | --- | --- |
| **Antibodies** |  |  |
| PIKfyve | Sigma | P0054 |
| β Actin | Abcam | Ab8224 |
| PIKfyve | Abnova | PAB17315 |
| VAC14 | Proteintech | 15771-1-AP |
| FIG4 | Santa Cruz | Sc-98633 |
| LAMP1 | Abcam | ab25630 |
| LAMP1 | Abcam | ab24170 |
| LAMP2 | Abcam | ab37024 |
| PERK | Cell Signaling | 3192S |
| (p)PERK | Cell Signaling | 3179S |
| eIF2α | Cell Signaling | 9722S |
| (p)eIF2α | Cell Signaling | 9721S |
| TRPML1 | Alomone Lab | ACC-081 |
| SARA | Abcam | ab221488 |
| GM130 | Thermofisher sceintific | PA5-95727 |
| Calnexin | Enzo Life sciences | ADI-SPA-865-D |
| GFP | abcam | ab290 |
| TFEB | Bethyl laboratories | MBS120432 |
| TFEB-pSer142 | Sigma Aldrich | ABE1971-I |
| Flag | Sigma Aldrich | A2220 |
| NeuN | abcam | ab104224 |
| Synaptophysin | abcam | ab32127 |
|  |  |  |
| **Other Reagents** |  |  |
| Alexa488-transferrin | Thermofisher | T13342 |
| L methionine+ L cysteine S35 | Hartmann Analytic | SCIS103 |
| 3H Palmitic acid | Hartmann Analytic | ART0129 |
| Alexa 647 Succinimidyl ester | Molecular probes | A20106 |
| Lysosensor blue DND | Thermofisher | L7533 |
| DMEM | Invitrogen | 41965-062 |
| Foetal bovine serum | Invitrogen | 10109-163 |
| Thapsigargin | Sigma Aldrich | T9033-1MG |
| RML6 prions | Lab | N/A |
| ME7 prions | Lab | N/A |
| Penicillin/Streptomycin | Invitrogen | 15140-130 |
| GlutaMax | Invitrogen | 35050-087 |
| GSK2606414 | Merck | 1337531-89-1 |
| ISRIB | Sigma Aldrich | SML0843-24MG |
| Sytox | Molecular probes | S7020 |
| DAPI | Sigma Aldrich | D9542 |
| Dynabeads Protein G | Invitrogen | 100-03D |
|  |  |  |
| **Cell lines** |  |  |
| Gt1 cells | Pamela Mellon Lab | N1A |
|  |  |  |
| **Commercial assays** |  |  |
| SyBr green PCR master mix | Applied biosystems | 4309155 |
| Beta Hexosaminidase assay kit | Cellbiolabs | MET5095 |
|  |  |  |
| **Recombinant DNA** |  |  |
| pCDNA3 | therrmofisher | V79020 |
| PIKfyve-GFP | Jeremy M Tavaré lab | N/A |
| zDHHC9-Flag | Sebastian Jessberger lab | N/A |
| zDHHC21-Flag | Sebastian jessberger lab | N/A |
| LV-GADD34 | Giovonna Mallucci lab | N/A |
| LV-Ctrl | Giovonna Mallucci lab | N/A |
|  |  |  |
| Software |  |  |
| Image J | NIH | https://imagej.nih.gov/ij/download.html |
| Graphpad Prism 8 | Graphpad | https://www.graphpad.com/scientific-software/prism/ |
| Quantitiy One | Bio-rad | https://www.bio-rad.com/en-ch/product/quantity-one-1-d-analysis-software?ID=1de9eb3a-1eb5-4edb-82d2-68b91bf360fb |
