# Supplementary material for: Loss of PIKfyve drives the spongiform degeneration in prion diseases
Source: EMBO Mol Med. 2021 Jul 22;13(9):e14714. doi: 10.15252/emmm.202114714 (PMC8518562; doi:10.15252/emmm.202114714)
Supplement: Supplementary file 5 — Table EV4 [file EMMM-13-e14714-s006.docx]

**Table EV4**: List of all siRNA used in the current study.

| Gene name | siRNA Target sequence (5´-3´) |
| --- | --- |
| Zdhhc1 | CCTGGTCCTAAAGGGATTA |
| Zdhhc2 | GGCACCATTTGCCAATTGT |
| Zdhhc3 | GGGCATAGAACAATTGAAA |
| Zdhhc4 | GTCCGACCTAGAGAAATAT |
| Zdhhc5 | GCCCAAGATTGAAGACAAA |
| Zdhhc6 | CTGGGTGTTATAGCAATAT |
| Zdhhc7 | GGGTGTTTCAGGGAATCAT |
| Zdhhc8 | CCTGCTCTATGTGCTCAAT |
| Zdhhc9 | GGCTCTTGATAATGTTTGA |
| Zdhhc11 | GTGCACTTGATCGCAATTA |
| Zdhhc12 | GGGAGTTCATATCTTCACA |
| Zdhhc13 | GCTGGTAGAAGCAGGATAT |
| Zdhhc14 | GAGGCTGTAATATGCTTCT |
| Zdhhc15 | GCAGGTGTTTGGCGATAAT |
| Zdhhc16 | GGCCATTGCTTATCTGTGT |
| Zdhhc17 | GCGACACAATATGGAATAT |
| Zdhhc18 | GGCAGACAGTGAAACTCAA |
| Zdhhc19 | GGGTCCCAATTACATGTCT |
| Zdhhc20 | GCCCTTCCAAAGAGTTCTA |
| Zdhhc21 | CCACCAGGGTTTCTTTAAA |
| Zdhhc22 | GCCCTTCTCTTGTGTTGAT |
| Zdhhc23 | GCGGGTTACTTCTGATACT |
| Zdhhc24 | GTGTGGGCTTCCATAATTA |
| PIKfyve | AAGGGTGAACTAGACAATA |
| FIG4 | TTCGACATCTTTGAAGATG |
| VAC14 | CAGACTGAAGACTGTCTGA |
| TFEB | GGCAGAAGAAAGACAATCA |
