# Supplementary figures and images for: Loss of PIKfyve drives the spongiform degeneration in prion diseases
Source: EMBO Mol Med. 2021 Jul 22;13(9):e14714. doi: 10.15252/emmm.202114714 (PMC8518562; doi:10.15252/emmm.202114714)

Appendix Figure S1

A

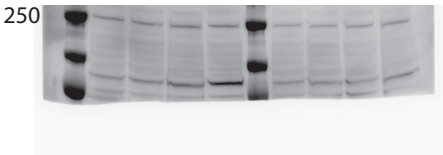

B

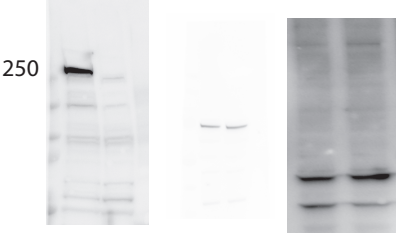

C

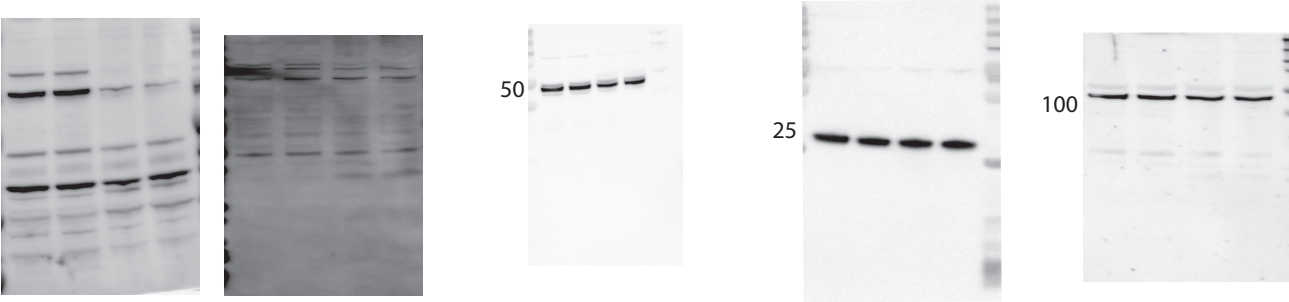

D

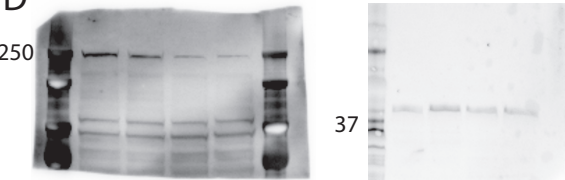

I

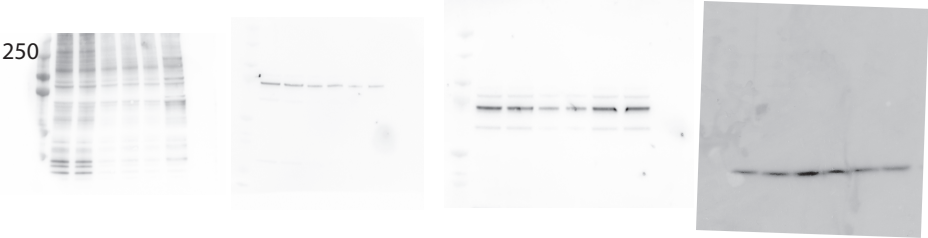

Supplement: Supplementary file 6 — Source Data for Appendix [file EMMM-13-e14714-s004.zip › Source_Appendix_Figures_pdf/Appendix Figure S1/EMM-2021_14714_Source data for Appendix Figure S1.pdf]

Appendix Figure S3

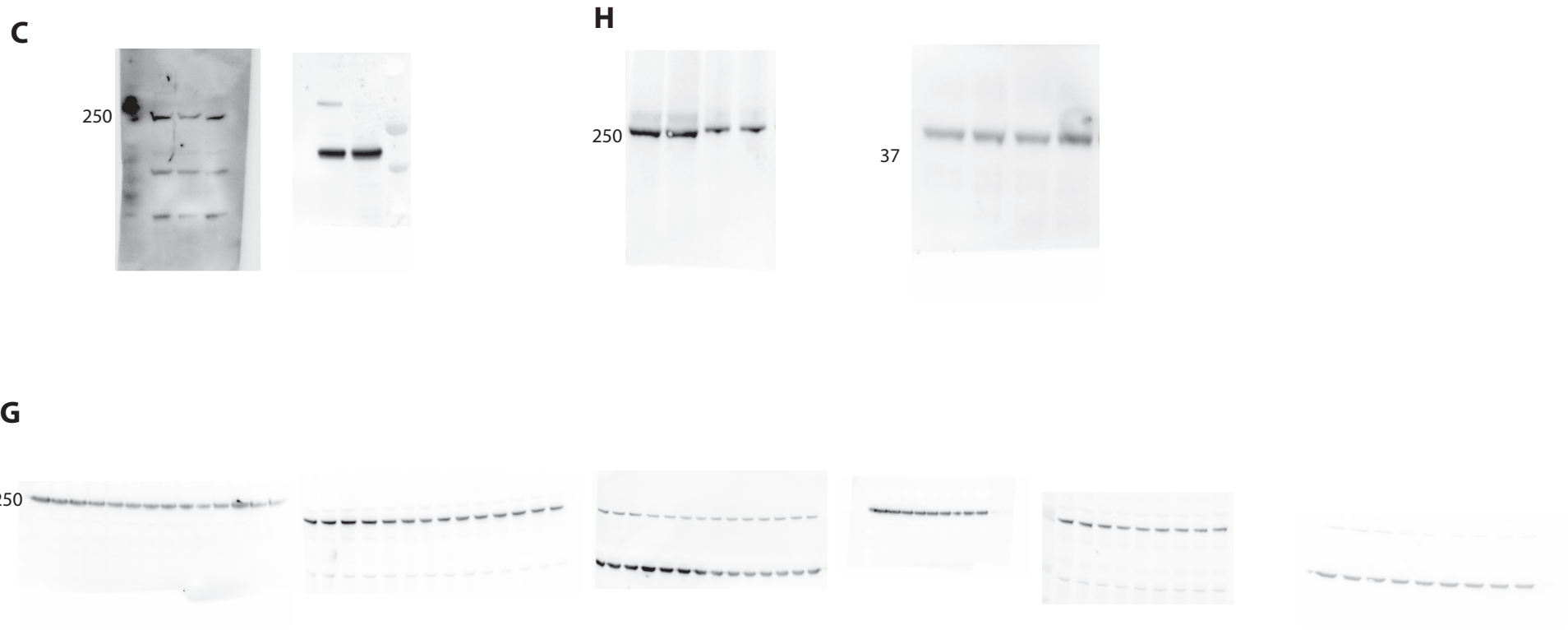

Supplement: Supplementary file 6 — Source Data for Appendix [file EMMM-13-e14714-s004.zip › Source_Appendix_Figures_pdf/Appendix Figure S3/EMM-2021_14714_Source data for Appendix Figure S3.pdf]

## Appendix Figure S2

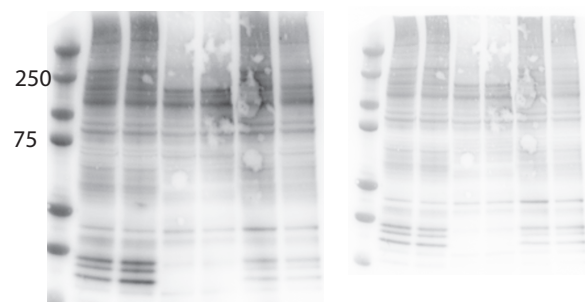

Supplement: Supplementary file 6 — Source Data for Appendix [file EMMM-13-e14714-s004.zip › Source_Appendix_Figures_pdf/Appendix Figure S2/EMM-2021_14714_Source data for Appendix Figure S2.pdf]

Figure1

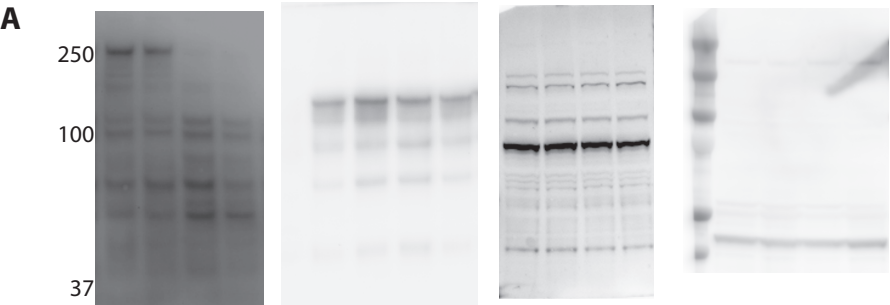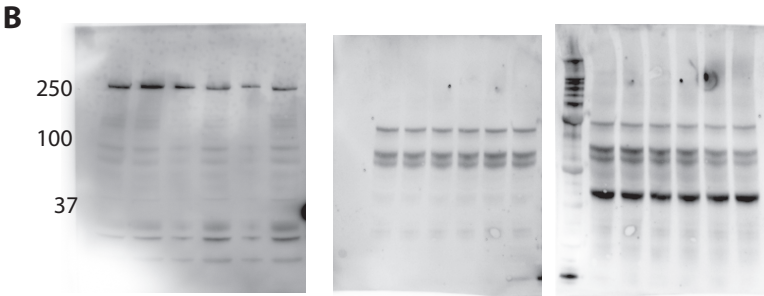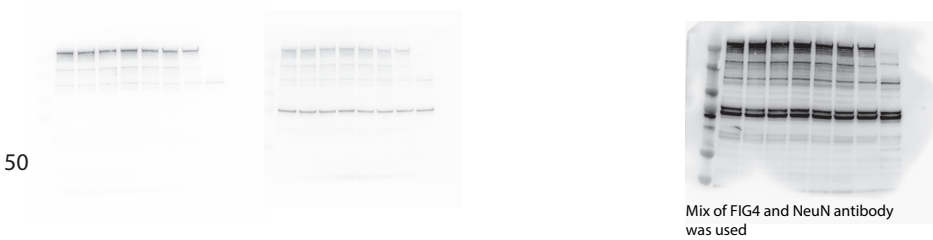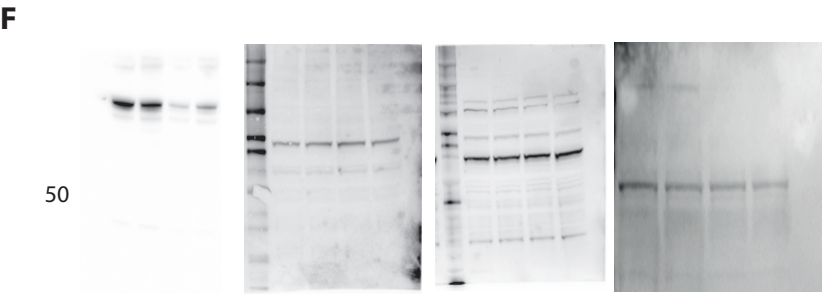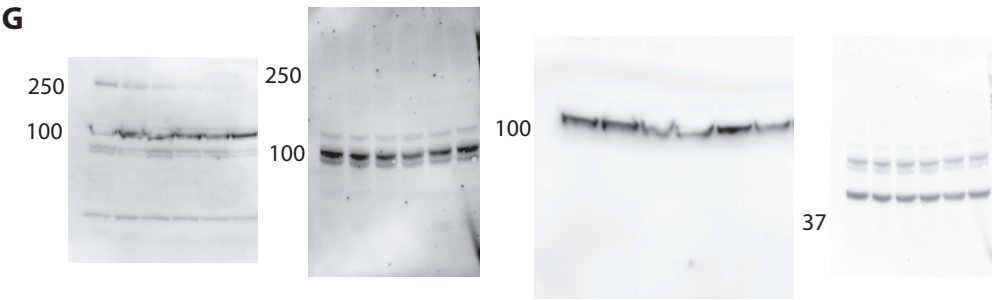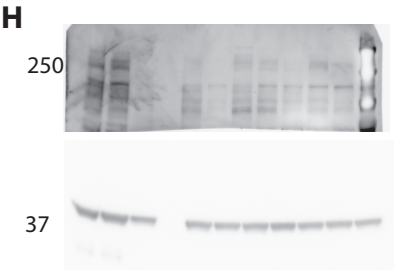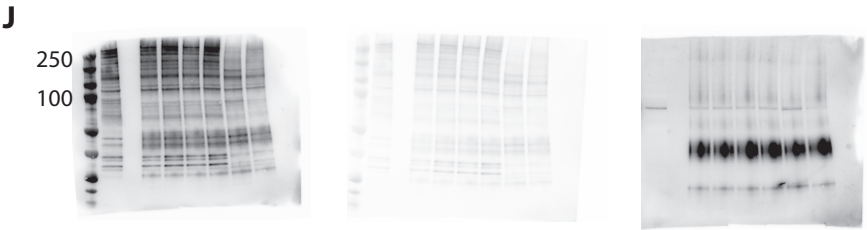

Supplement: Supplementary file 7 — Source Data for Figure 1 [file EMMM-13-e14714-s009.pdf]

Figure 2

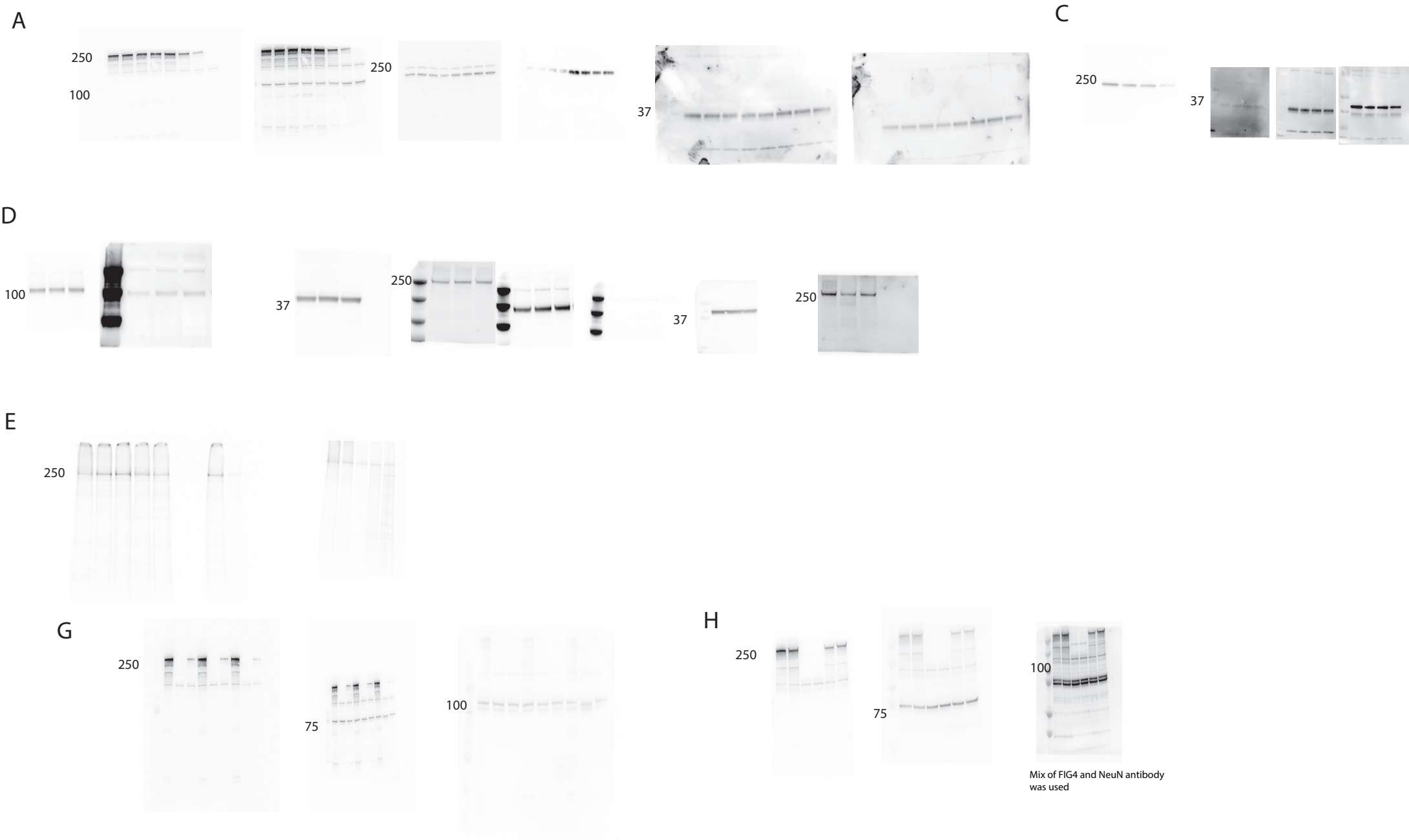

Supplement: Supplementary file 8 — Source Data for Figure 2 [file EMMM-13-e14714-s002.pdf]

**Figure 3**

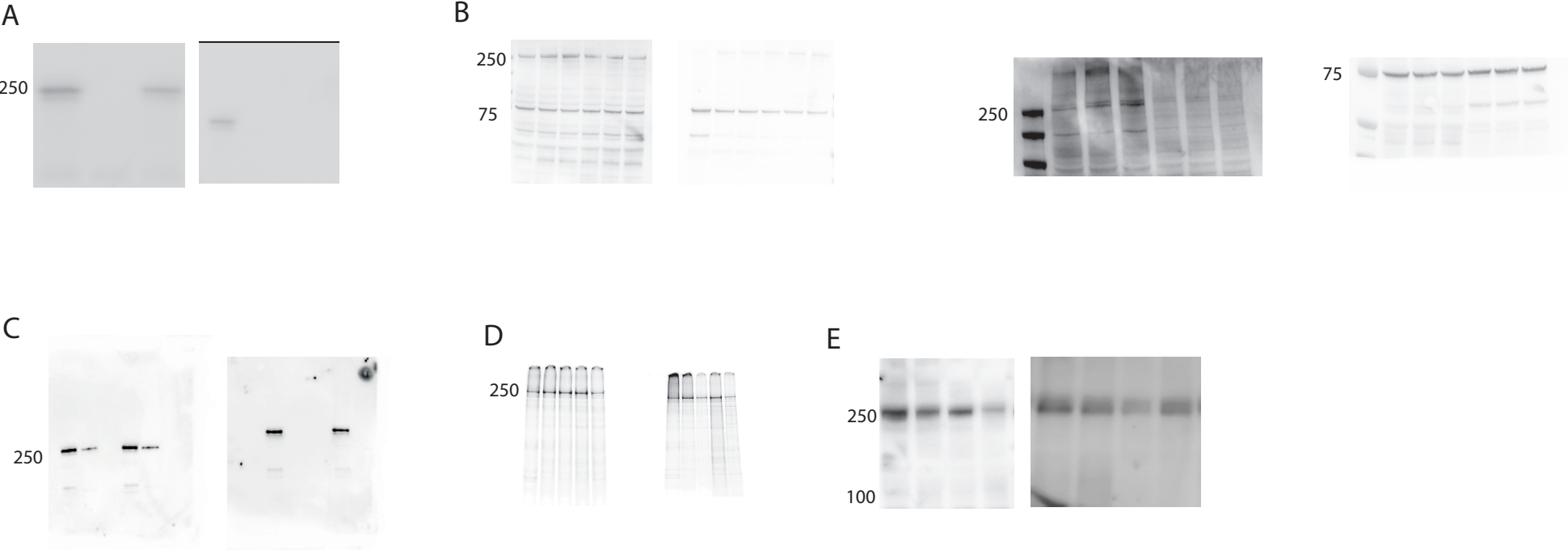

Supplement: Supplementary file 9 — Source Data for Figure 3 [file EMMM-13-e14714-s011.pdf]

Figure 5

A

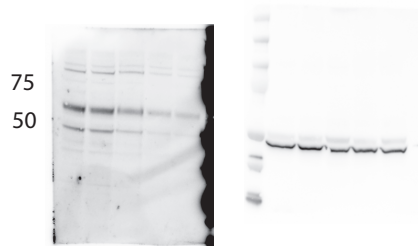

C

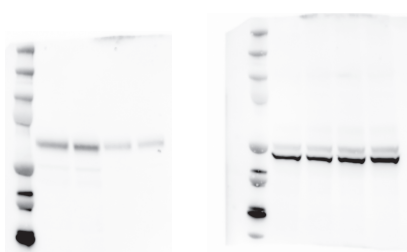

D

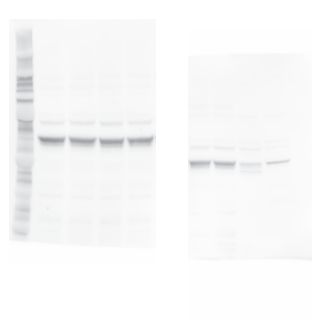

Supplement: Supplementary file 10 — Source Data for Figure 5 [file EMMM-13-e14714-s001.pdf]

**Figure 6**

**A**

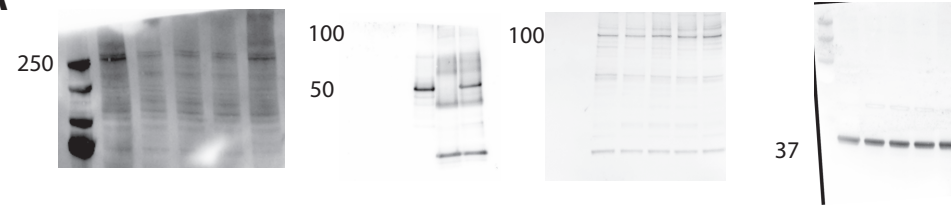

**C**

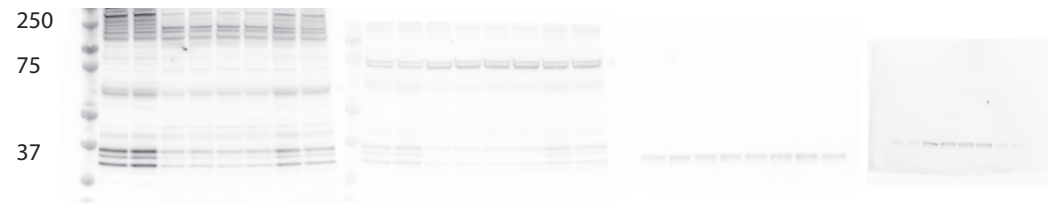

Supplement: Supplementary file 11 — Source Data for Figure 6 [file EMMM-13-e14714-s007.pdf]
